# Blocking mitochondrial leucine transamination enhances T-cell activation and improves T-cell immunity against OVA-producing EL4 lymphoma

**DOI:** 10.1038/s41416-026-03455-5

**Published:** 2026-05-05

**Authors:** Christie M. Adam, Tanner J. Wetzel, Sheila C. Erfan, Leighton M. Wheeler, Emily E. Baer, Lindsey P. Croll, Alexander M. Martin, Taha Z. Khan, Max L. Swain, Michael P. Boyer, Elitsa A. Ananieva

**Affiliations:** 1 Department of Biochemistry and Nutrition, Des Moines University, West Des Moines, IA USA; 2 UF Shands Hospital, University of Florida, Gainesville, FL USA; 3 Flushing Hospital Medical Center, New York, NY USA

**Keywords:** Cancer metabolism, Tumour immunology

## Abstract

**Background:**

T-cell metabolism is targeted by cancer cells in an attempt to escape immune surveillance. The mitochondrial branched-chain aminotransferase, BCATm, is overexpressed in cancer, yet its role in T-cell immunity is suggested but understudied.

**Methods:**

C57BL/6 mice with T-cell specific-single BCATm deficiency were used to determine the impact of BCATm on T-cell function in vitro and in vivo using the murine EL4-OVA lymphoma. The studies were complemented by a transcriptomic correlation analysis of BCATm in human T cells and by using siRNA to knock-down BCATm in Jurkat T cells.

**Results:**

The loss of BCATm from CD4^+^ T cells increased mitochondrial respiration but reduced the coupling between oxygen consumption and ATP synthesis, redirecting the cells to glycolysis. This compensation sustained T-cell functionality as seen by increased release of IFN-γ from CD4^+^ T cells or granzyme B and perforin from CD8^+^ T cells. Human studies further suggested that BCATm negatively affected T-cell mitochondria. While EL4-OVA tumours from T-BCATm^KO^ mice were enriched in memory precursor CD4^+^ and CD8^+^ T cells, reduced EL4-OVA lymphoma growth was achieved in mice with T cells carrying a combined deletion of BCATm and BCATc.

**Conclusions:**

BCATm is an immunosuppressive enzyme that may weaken T-cell performance in the lymphoma microenvironment.

## Background

Increased aerobic glycolysis and uptake of amino acids are hallmarks of activated T cells that afford continuous supply of metabolites for anabolic processes [1, 2]. Despite these metabolic adaptations, T cells experience functional defects and enter a state of exhaustion in the tumour microenvironment (TME), due to tumour-enforced metabolic barriers including the buildup of immunosuppressive metabolites [3]. Moreover, terminally-exhausted T cells, while exhibiting glycolytic phenotype, are unable to self-renew in the presence of chronic antigen stimulation due to impaired oxidative phosphorylation [4].

The mitochondrial branched-chain aminotransferase, BCATm, specialises in the reversible transamination of the branched-chain amino acids (BCAAs) leucine, isoleucine, and valine to their corresponding branched-chain α-keto acids [5]. This evolutionary conserved enzyme requires α-ketoglutarate as the α-amino group acceptor, which is converted into glutamate during catalysis. This property is shared by a diverse group of aminotransferases [6, 7]. BCATm works near the mitochondrial branched-chain α-keto acid dehydrogenase (BCKDH) complex that commits BCAAs to irreversible oxidation [8]. While oxidation of BCAAs is downregulated in cancerous tissues, BCATm-driven mitochondrial transamination supports de novo nucleotide synthesis via the glutamine-arginine axis as shown in non-small-cell lung cancer (NSCLC) [9].

BCATm, together with its cytosolic isoenzyme, BCATc, is frequently found overexpressed in cancer, but their roles in the interplay between cancer and immune cells is poorly understood [10, 11]. Recent studies suggested that the BCAT isoenzymes have immunoregulatory properties [12–15]. This, taken together with the growing interest in T-cell mitochondria [16, 17], raised the question whether BCATm has an uncovered role in controlling the function of T-cell mitochondria. The objective of this report was to investigate how mitochondrial leucine transamination via BCATm affects the metabolic reprogramming and function of activated T cells and their ability to fight lymphoma using murine models and human T cells. Our findings revealed a novel immunosuppressive role of BCATm in T cells in vitro and during a challenge with EL4-OVA lymphoma.

## Methods

### Pharmaceutical chemicals and antibodies

N-acetyl-leucine amide (NALA), a competitive antagonist of leucine, was purchased from Bachem (Bubendorf, Switzerland, Cat # E-1105) to mimic leucine depletion. BCAT-IN-2, a selective inhibitor of BCATm, was purchased from MedChem Express, US (Cat # HY-141669). Control-siRNA and two clones of *BCAT2*-siRNAs were purchased from Horizon Discovery (Cambridge, UK) to target the human *BCAT2* gene (encodes BCATm) in Jurkat T cells. Information regarding antibodies is in Supplementary Table 1.

### Human gene expression datasets and analysis

The genomics and visualisation platform R2 (http://r2.amc.nl) was used for the acquisition of human RNA-seq datasets aiming at comparing *BCAT2* and in some of the experiments *BCAT1* (the gene encoding BCATc) in T cells from healthy donors and patients with peripheral T-cell lymphoma (PTCL), anaplastic T-cell lymphoma (ATCL), angioimmunoblastic T-cell lymphoma (AITL) or T-cell acute lymphoblastic leukaemia (T-ALL). A dataset by Eckerle et al. [18] contained gene expression profiles of resting CD4^+^ (*n* = 5) and CD8^+^ (*n* = 5) T cells and activated CD4^+^ (*n* = 5) and CD8^+^ (*n* = 6) T cells isolated from tonsils of healthy human donors. This dataset was used to compare the expression of *BCAT2* between resting (*n* = 10) and activated (*n* = 11) T cells by using the “singular gene versus track” analysis of R2. Resting and activated T-cell groups were prepared by pooling the CD4^+^ and CD8^+^ T cells of each group due to the small sample size; however, the individual trends of these cells were similar. The activated T cells were subjected to KEGG pathway analysis generated in the R2 platform with the following specifications: the “KEGG pathway finder for gene correlation” analysis was selected, followed by identifying the gene reporter (*BCAT2*) using transformation Log2, and correlation R that included positive and negative gene correlations and correlation *p* value cutoff < 0.05. The first five KEGG pathways, and associated genes are shown in Supplementary Tables 2 and 3. A T-cell lymphoma/leukaemia dataset deposited by Crescenzo et al. [19] included gene expression profiles from in vitro activated CD4^+^ (*n* = 4) and CD8^+^ (*n* = 4) T cells obtained from healthy individuals or patients with PTCL (*n* = 35), ATCL (*n* = 23), AITL (*n* = 24) and T-ALL (*n* = 4) [19] The gene information of this dataset was analysed by sorting the specimens by cell type and disease status. To explore the differential expression of *BCAT2* and *BCAT1*, a “singular gene versus track” analysis was performed.

Correlation between the expression of *BCAT2, or BCAT1*, and the overall survival of patients with T-cell lymphoma was performed with the Kaplan–Meier Scanner of R2 using a “single gene versus track” analysis. The R2 platform contained only one T-cell lymphoma study (*n* = 193) with survival data for female (*n* = 55) and male (*n* = 92) patients with PTCL [20]. The minimum group size was set at *n* = 8 with follow-up to 200 months. The data was transformed into the Log2 scale and represented as percentage of mixed sex, averaged, and subjected to One-way ANOVA where *p*  <  0.05 was considered statistically significant.

### Mice

**Fig. 1:**
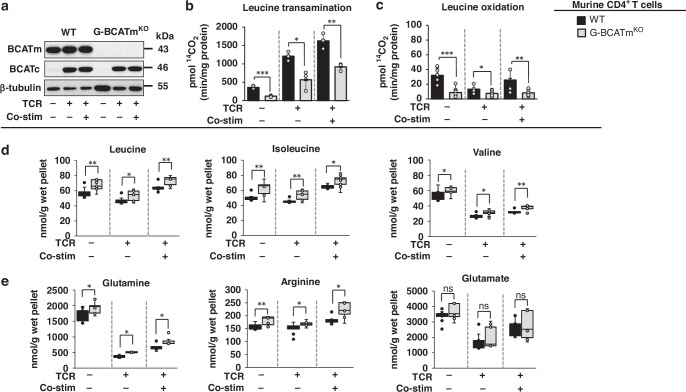
A loss of BCATm expression from T cells reduces BCAA catabolism but increases the intracellular levels of glutamine and arginine. **a**–**e** Previously activated CD4^+^ T cells from WT and global BCATmKO (G-BCATm^KO^) mice were left unstimulated [-] or re-challenged with anti-CD3 (TCR or anergic [+/-]), or anti-CD3/CD28 (co-stimulated, full activation [+/+]) for 24 h, *n* = 18 mice/variant, mixed sex. Material from these cells was used to determine the expression of BCATm and its cytosolic isoenzyme BCATc (**a**) the rates of leucine transamination and oxidation (**b**, **c**) and the intercellular concentrations of leucine, isoleucine, and valine (**d**) or glutamine, arginine and glutamate (**e**). In all graphs, data represent mean ± SEM or ± SD of 3 independent experiments each with *n* = 2–3 pooled mouse spleens/lymph nodes/variant. Statistical significance as determined by the two-tailed Student’s t test: *p ≤ 0.05, **p ≤ 0.01, ***p ≤ 0.001, or ns no significance as compared to WT T cell variants. In **a** western blotting image is representative of 3 independent experiments.

**Fig. 2:**
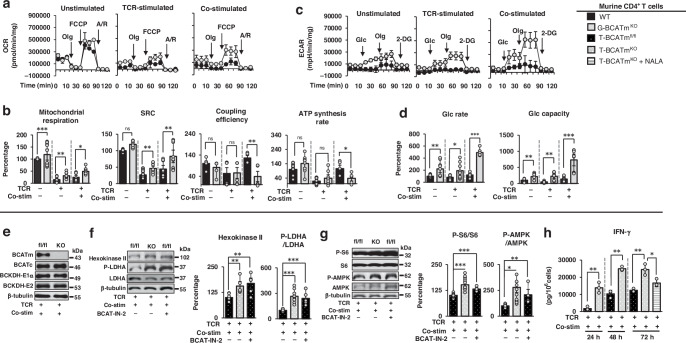
A loss of BCATm from co-stimulated murine CD4^+^ T cells leads to mitochondrial uncoupling and high glycolytic flux. **a**–**d** Previously activated CD4^+^ T cells from WT and G-BCATm^KO^ mice were left unstimulated [-] or re-challenged with anti-CD3 (TCR or anergic [+/-]), or anti-CD3/CD28 (co-stimulated [+/+]) for 24 h, *n* = 3 independent experiments each with *n* = 2–3 biological replicates, mixed sex. Material from these cells was used to measure oxygen and glycolytic metabolism by using the XF24 flux analyser (Agilent, see Methods). In **a**, **c** average oxygen consumption rate (OCR) and extracellular acidification rate (ECAR) in unstimulated, TCR- stimulated, or co-stimulated WT and G-BCATm^KO^ CD4^+^ T cells. In **b**, **d**, average mitochondrial respiration, spared respiratory capacity (SCR), coupling efficiency, ATP synthesis rate, basal glycolytic (Glc) rate, and glycolytic (Glc) capacity calculated as described under Methods. **e**–**h** Previously activated CD4^+^ T cells from T-cell conditional knockout of BCATm (T-BCATm^KO^ [KO]) and floxed control T-BCATm^fl/fl^ [fl/fl] mice, were re-challenged with anti-CD3/CD28 (co-stimulation [+/+]) for 24 h, *n* = 3 independent experiments each with *n* = 2–3 biological replicates, mixed sex. Some of the cells from T-BCATm^fl/fl^ mice were re-challenged in the presence of 50 μM BCAT-IN-2 to inhibit the enzyme activity of BCATm (**f**, **g**). The expression and the phosphorylation (P) states (LDHA, S6 and AMPK only) of the following proteins were determined via western blotting: BCATm, BCATc, BCKDH-E1α, and BCKDH-E2 (**e**) hexokinase II and LDHA (**f**) and S6 and AMPK (**g**). In **h** ELISA was done on supernatants from co-stimulated cells to determine the secretion of IFN-γ. Some of the T-BCATm^KO^ T cells (72 h) were pre-treated with 20 mM NALA to mimic leucine depletion. In **f**, **g** Image J was used to quantify the relative band intensity of hexokinase II as normalised to β-tubulin, while P-LDHA, P-S6, and P-AMPK were normalised to LDHA, S6, and AMPK, respectively, and presented as percentage of littermate control or untreated cells. Western blot images were representative of 3 independent experiments or 2 or more sets of 3 mice/variant. For all panels, average +/- SEM or +/-SD of mixed sex. Statistical significance as determined by the two-tailed Student’s *t* test: *p ≤ 0.05, **p ≤ 0.01, ***p ≤ 0.001 or ns no significance. Abbreviations: AMPK AMP-activated protein kinase, A/R antimycin and rotenone, 2-DG 2-deoxy-D glucose, FCCP carbonyl cyanide p-trifluoromethoxyphenylhydrazone, Glc glucose, LDHA lactate dehydrogenase, Olg oligomycin, S6 ribosomal S6 protein.

Mice from the C57BL/6 background were approved by the Institutional Animal Care and Use (IACUC) Committee of Des Moines University. The global BCATmKO (G-BCATm^KO^) and wild-type (WT) mice were maintained at Virginia Tech [15]. They were a source of CD4^+^ T cells in Figs. 1–2. Mice with loxP-flanked *Bcat2* or *Bcat1* alleles were generated previously [21, 22]. CD4-Cre mice from the Jackson laboratory (strain # 017336) were bred with mice carrying the loxP-flanked *Bcat2* and *Bcat1* alleles to generate single (BCATm or BCATc) and double (BCATc-BCATm) deletions in single and double positive CD4^+^ and CD8^+^ T cells of the newly established T-BCATm^KO^, T-BCATc^KO^ and T-Bc^KO^Bm^KO^ mouse lines. As control littermates, T-BCATm^fl/fl^, T-BCATc^fl/fl^ and T-Bc^fl/fl^Bm^fl/fl^ mice carrying the loxP-flanked *Bcat2* or *Bcat1* or the combination of the loxP-flanked *Bcat1* and *Bcat2* alleles in homozygous states were used. PCR genotyping was performed on mouse ear genomic DNA using primers shown in Supplementary Table 4. Additional details are in the Supplementary information (Sections 15–16).

### Cancer cell lines

Human Jurkat T cells (clone E6-1, Cat # TIB-152) and the murine EG7 (EL4-OVA, Cat # CRL-2113) cells expressing chicken ovalbumin (OVA), were purchased from ATCC (Manassas, VA). The cells were maintained in RPMI-1640 medium (CellGro, Corning, NY) supplemented with 10% FBS, 100 IU/ml penicillin, 100 μg/ml streptomycin and 0.05 mM β-ME (EL4-OVA) in a tissue culture incubator at 37 °C and 5% CO_2_. The OVA antigen was maintained in the presence of 0.04 mg/ml G418. Cells were allowed 1–3 passages, free of mycoplasma, and their identity was confirmed by short tandem repeats (STR) analysis (LabCorp, Burlington, NC). Refer to Supplementary information (Section 16) for more details.

### In vivo tumour mouse studies

Male and female T-BCATm^fl/fl^, T-BCATm^KO^, male T-BCATc^fl/fl^, T-BCATc^KO^, and male T-Bc^fl/fl^Bm^fl/fl^ and T-Bc^KO^Bm^KO^ mice, age 8–15 weeks, received s.c. injections of 2.5 × 10^5^ EL4-OVA cells in 100 μl sterile 1X PBS buffer on the lower back. Vehicle controls received 100 μl sterile 1X PBS buffer. Mice were caged individually, and food intake and body weight were measured daily. Tumour volumes were measured daily with a digital caliper (formula: length × (width)^2^/2) until day 15. Tumours and organs were weighed and stored at −80 °C. Additional details are in the Supplementary information (sections 1–8).

### T-cell isolation, expansion, and activation

Spleens and lymph nodes from WT, G-BCATm^KO^, T-BCATm^fl/fl^, T-BCATm^KO^, T-Bc^fl/fl^Bm^fl/fl^ and T-Bc^KO^Bm^KO^ mice, were pooled to increase T-cell yield (*n* ≥ 2–3 spleens/variant) and passed through a 70–100 µm strainer followed by lysis with ACK buffer to remove red blood cells. CD4^+^ T cells were isolated via negative magnetic separation using a CD4^+^ T-cell isolation kit (Miltenyi Biotec, Gaithersburg, Maryland) and activated with 2 µg/ml anti-CD3/anti-CD28 for 48 h and expanded with 20 ng/ml IL**-**2 for 2–3 days as described [21]. The cells were left unstimulated or stimulated with anti-CD3 (T-cell receptor [TCR]-stimulated, or also referred to as anergic), or anti-CD3/anti-CD28 (co-stimulated or also referred to as activated) for 24–72 h. Some cells were simultaneously treated with 20 mM NALA or 50 µM BCAT-IN-2.

CD8^+^ T cells were isolated via negative magnetic separation using a CD8^+^ T cell isolation kit (Miltenyi Biotec, Gaithersburg, Maryland) and stimulated with 3 µg/ml anti-CD3/anti-CD28 for 48 h. Mouse IL-2 (20 ng/ml) and IL-7(5 ng/ml) were added 24 h post activation. This is referred to as the “activation phase”. After 48 h, 1 × 10^6^ cells were sub-cultured for an additional 48 h (96 h expansion), without antibodies, but in the presence of IL-2 and IL-7 (referred to as the “expansion phase”). Following these phases, cells were collected, washed twice in ice-cold 1X PBS buffer, pelleted at 13,200 rpm for 7 min at 4 °C, and stored at −80 °C along with collected supernatants.

### Leucine oxidation and transamination assay

Previously experienced, unstimulated, TCR-stimulated, or co-stimulated CD4^+^ T cells from WT and G-BCATm^KO^ mice, or CD4^+^ T cells from T-Bc^fl/fl^Bm^fl/fl^ and T-Bc^KO^Bm^KO^ mice, activated with 2 µg/ml anti-CD3/anti-CD28 for 48 h, or untreated Jurkat T cells, were incubated in Krebs buffer in the presence of ^14^C-leucine to determine the rates of leucine oxidation and transamination as described [21]. Results were represented as picomoles of released ^14^CO_2_/min/mg protein.

### Amino acid analysis

Amino acid concentrations in cell pellets (nmol/g wet pellet) and supernatants (μM) from previously experienced, unstimulated, TCR-stimulated, or co-stimulated CD4^+^ T cells from WT and G-BCATm^KO^ mice were measured by HPLC after ophthaldialdehyde derivatization on a Supelcosil™ LC-18 column (15 cm × 4.6 mm, 3 μm) (Sigma, St. Louis, MO) as described [21].

### Measurements of glucose and oxygen metabolism

Glucose and oxygen metabolism were measured in previously experienced, unstimulated, TCR-stimulated, or co-stimulated CD4^+^ T cells from WT and G-BCATm^KO^ mice. The cell preparation and the measurement of glucose metabolism were described [21]. Oxygen metabolism was determined in the same variants with the following specifications. XF24 flux analyser (Agilent, Santa Clara, CA) was used to measure the oxygen consumption rate (OCR) in the presence of oligomycin (Olg,1 µM) to inhibit ATP synthesis, the uncoupler FCCP (1.5–2 µM) of the oxidative phosphorylation, and antimycin and rotenone (A/R, 0.5–1 µM) to inhibit the electron transport chain (ETC). Mitochondrial respiration was calculated by subtracting the non-mitochondrial OCR from the FCCP-OCR. The spared respiratory capacity (SRC) was calculated by subtracting the basal-OCR from the maximal respiration rate after injecting FCCP. OCR-ATP (oxygen consumed for ATP production) was calculated by subtracting OCR after oligomycin from the basal-OCR. The mitochondrial ATP production was calculated by the formula: mitoATP rate = OCR-ATP × 2 × 2.75 (2.75 is the phosphorus/oxygen ratio, Agilent software). The coupling efficiency was calculated by the formula: (ATP production rate)/(basal respiration rate) x 100%. The glycolytic rate, glycolytic capacity, mitochondrial respiration, SRC, mitochondrial ATP production, and coupling efficiency were normalised against the unstimulated WT T cells followed by calculation of percentage difference between T cells from G-BCATm^KO^ and WT mice for the remaining variants.

### Western blotting assay

Protein from murine CD4^+^ and CD8^+^ T cells, or human Jurkat T cells, or mouse tissues, was subjected to BCA protein assay (Thermo Scientific, Waltham, MA) followed by western blotting as described [21]. Protein bands detected using a developer and x-ray films were photographed, and band intensities quantified with Image J [23]. Protein bands corresponding to BCATc, BCATm, BAX, BCKDH-E1α, BCKDH-E2, Hexokinase II, NDUFSI, CD244, cytochrome c, COX IV, LAG3, TOX, TIM-3, TIGIT, TCF1/7, were normalised to β-tubulin or GAPDH, while those corresponding to S6, P-S6, AMPK, P-AMPK, LDHA, P-LDHA, Rb, P-Rb were represented as the ratio between the phosphorylated (P) and total concentrations of S6, AMPK, LDHA, or Rb, respectively. The antibodies are listed in Supplementary Table 1. Results are shown as a percentage of the corresponding control.

### Flow cytometry

Spleens, lymph nodes, and thymuses from T-BCATm^fl/fl^ and T-BCATm^KO^ mice, were homogenised and subjected to ACK lysis (spleens only) as described above. EL4-OVA tumours from T-BCATm^fl/fl^ and T-BCATm^KO^ mice were dissociated using the tumour dissociation kit (Miltenyi Biotec, Gaithersburg, Maryland) and the gentle MACs OctoDissociator following the manufacturer’s recommendations. 1 × 10^6^ cells/variant were stained with fluorescently-conjugated antibodies targeting CD4, CD8, CD62L, CD127, and KLRG1 in ice-cold stain buffer (BD Pharmingen, Cat # 554656) for 30 min in the dark. Flow cytometry was performed using the Attune NxT acoustic focusing flow cytometer (Thermo Scientific, Waltham, MA) and results were analysed with FlowJo v10. The antibodies are listed in Supplementary Table 1. The gating strategy is shown in Supplementary Fig. 1.

### IFN-γ, perforin, and granzyme B, ELISA assays

Supernatants from previously experienced and co-stimulated (24–72 h) T-BCATm^fl/fl^ and T-BCATm^KO^ CD4^+^ T cells or T-BCATm^fl/fl^ and T-BCATm^KO^ CD8^+^ T cells were subjected to ELISA to determine the extracellular secretion of IFN-γ (CD4^+^ T cells, Tonbo BioSciences, San Deigo, CA), perforin (CD8^+^ T cells, Novus Biologicals, Centennial, CO) or granzyme B (CD8^+^ T cells (Thermo Scientific, Waltham, MA) by following the manufacturer instructions. Results are shown as picograms (pg) of secreted cytokine/1×10^6^ cells, or nanograms (ng) of secreted cytokine/mg protein.

### Cytotoxic killing assay

EL4-OVA cells (10 × 10^6^/reaction) were transfected with 0.4 μg pRL Renilla luciferase vector (Promega, Madison, WI, Cat # E2231), via electroporation (Bio-Rad Gene Pulser, Bio-Rad, Hercules, CA), prior to co-culturing with expanded T-BCATm^fl/fl^ or T-BCATm^KO^ CD8^+^ T cells. Electroporated, but un-transfected, or Renilla-transfected, but cultured alone, EL4-OVA cells served as un-transfected and transfected controls, respectively. EL4-OVA and CD8^+^ T cells were co-cultured at a ratio of 1:2.5 and incubated in T-cell medium for 7 h followed by measurement of luminescence released by the EL4-OVA cells. Luminescence was detected using the dual luciferase reporter assay following the manufacturer instructions (Promega, Madison, WI, Cat # E1910).

### siRNA transfection and electroporation

Jurkat T cells (10 × 10^6^/variant) were washed with ice-cold 1X PBS buffer and resuspended in 1 M transfection buffer (5 mM KCI, 15 mM MgCI_2_, 120 mM Na_2_HPO_4_/NaH_2_PO_4_, pH 7.2, 50 mM mannitol) in the presence of 200 nM control-siRNA or *BCAT2*-siRNA #1 or #2. Cells were subjected to electroporation to allow entry of siRNAs and left on ice for 30 min before transferring to 25 cm^2^ flasks supplemented with RPMI-1640 in the absence of FBS or antibiotics for 24 h. After 24 h, fresh media with 10% FBS was added, and cells were incubated for an additional 48 h. At the end of the siRNA transfection (72 h), cells were used in assays or washed twice in ice-cold 1X PBS buffer, pelleted at 13,200 rpm for 7 min at 4 °C, and stored at −80 °C.

### Quantitative RT-qPCR

Total mRNA was extracted from CD4^+^ T cells of T-Bc^KO^Bm^KO^ mice and littermate controls using the SV total RNA isolation kit (Promega, Madison WI) followed by cDNA synthesis using GoTaq 2-Step RT-qPCR kit (Promega, Madison WI). cDNA was quantified using Nanodrop8000 (Thermo Scientific, Waltham, MA). RT-qPCR was performed utilising the Biorad iTAQ Universal SYBR green super mix (Thermo Scientific, Waltham, MA). Primers used to amplify *Bcat1*, *Bcat2* and the eukaryotic translation elongation factor 1-alpha (*Ef1α)* (internal control) are in Supplementary Table 4. Fold changes in the expression of *Bcat1* and *Bcat2* were analysed using the ΔΔCt method and normalised to the expression of *Ef1α*. Results were presented as fold difference from CD4^+^ T cells of littermate controls.

### Cell cycle assay

Previously experienced CD4^+^ T cells from T-BCATm^fl/fl^ and T-BCATm^KO^ mice were co-stimulated for 24–48 h and fixed with 66% ethanol for up to 7 days followed by incubation in 50 mg/ml PI and 100 mg/ml RNase A staining solution for 30 min in the dark as described [21]. FACS (Becton, Franklin Lakes, NJ) was used to monitor the progression of the cells through the cell cycle.

### Cell viability assay

Jurkat T cells (10 × 10^6^/variant, *n* = 3 independent cultures with *n* = 3 technical replicates) transfected with control siRNA or *BCAT2*-siRNA (#1 or #2) were incubated with 0.5 mg/mL MTT (Tocris, Bristol, UK) for 1 h at 37 °C until it was reduced to an insoluble formazan. Formazan was solubilized with DMSO, and the absorbance of the resultant purple colour was read at 570 and 620 nm using a plate reader. Cell viability was calculated as a percentage from control cells.

### Cell proliferation assay

Jurkat T cells (10 × 10^6^/variant, *n* = 3 independent cultures with *n* = 3 technical replicates) transfected with control-siRNA or *BCAT2*-siRNA (#1 or #2) were permeabilized to allow for uptake of Ki67 or control (IgG isotope) antibodies as guided by the Muse Ki67 proliferation kit (Luminex, Austin, TX, cat# MCH100114). Proliferation was assessed using the Guava Muse cell analyser (Luminex, Austin, TX) by following the manufacturer recommendations. The percentage of undivided and divided Ki67 cells was normalised to IgG isotope treated cells.

### Statistical analysis

A two-tailed Student’s *t* test was used to determine statistically significant differences between each two groups of untreated (or in some experiments, NALA or BCAT-IN-2 -treated) CD4^+^ or CD8^+^ T cells from WT and G-BCATm^KO^ mice, or T-BCATm^fl/fl^ and T-BCATm^KO^ mice, or spleen, lymph nodes, thymus, or tumours from T-BCATm^fl/fl^ and T-BCATm^KO^ mice, or T-Bc^fl/fl^Bm^fl/fl^ and T-Bc^KO^Bm^KO^ mice (tumours only), or Jurkat T cells. One-way ANOVA was used to determine statistically significant differences between vehicle or OVA-inoculated male and female T-BCATm^fl/fl^ and T-BCATm^KO^ mice, or male T-BCATc^fl/fl^ and T-BCATc^KO^ mice, or T-Bc^fl/fl^Bm^fl/fl^ and T-Bc^KO^Bm^KO^ mice, over time. The in vitro data was analysed as a mixed sex (mouse T cells). Values were presented as mean ± SEM when 3 or more independent experiments or biological replicates were averaged or ± SD when a single representative experiment was shown. Each individual assay was run in *n* = 3–9 technical replicates/variant. The number of experiments or biological replicates is indicated in the figure legends. *P* ≤ 0.05 was considered statistically significant.

## Results

### BCATm supports mitochondrial leucine transamination in CD4^+^ T cells

Naïve CD4^+^ T cells break down glucose, amino acids, and lipids to meet basic metabolic needs [24, 25]. Consistent with this notion, BCAAs were subjected to BCATm-dependent mitochondrial breakdown in unstimulated WT CD4^+^ T cells (Fig. 1a–c). When these cells entered a state of anergy (TCR-stimulated cells) or activation (co-stimulated cells), they continued to rely on BCATm; however, TCR-engagement and co-stimulation of T cells led to the induction of BCATc to control the large cytosolic influx of leucine following T-cell activation (Fig. 1a) [21]. When BCATm was knocked out in CD4^+^ T cells, leucine transamination and oxidation were significantly reduced (Fig. 1b, c). This was accompanied by significant increases in the intracellular concentrations of leucine, isoleucine, and valine (Fig. 1d). Because BCATc was expressed in TCR- and co-stimulated cells, the residual BCAT metabolic activity in the absence of BCATm was attributed to BCATc (Fig. 1a–d). In summary, BCATm is constitutively expressed in murine T cells affording mitochondrial transamination of BCAAs regardless of the activation state.

### Co-stimulated CD4^+^ T cells deficient in BCATm have an increased uptake and intracellular concentrations of glutamine and arginine

To understand how a loss of BCATm may affect the rest of the amino acids, their extracellular and intracellular concentrations were measured in all variants (Supplementary Tables 5 and 6). Most of the extracellular amino acid concentrations were lower in the growth medium of TCR- and co-stimulated CD4^+^ T cells following 24 h growth, which reflected on the increased metabolic activity upon stimulation (Supplementary Table 5, compare 0 h and 24 h). Of note, the cells were grown in medium enriched in glutamine (2.7 mM, Supplementary Table 5). Following 24 h growth, TCR- and co-stimulated cells, but not unstimulated cells, from WT or G-BCATm^KO^ mice, had significantly lower extracellular glutamine suggesting an increased uptake upon stimulation (Supplementary Table 5). The highest uptake of glutamine (a 31% increase at 24 h) was recorded for co-stimulated cells from G-BCATm^KO^ mice (Supplementary Table 5). The intracellular glutamine in these cells was ~57% lower when compared to unstimulated cells (Fig. 1e, grey bars for glutamine and Supplementary Table 6) confirming an increased metabolic demand for glutamine upon T-cell activation. However, co-stimulated cells of G-BCATm^KO^ mice had significantly more intracellular glutamine (27%) compared with T cells of WT mice (Fig. 1e). A similar increase (~25%) was observed for arginine (Fig. 1e). No changes due to the loss of BCATm were observed for glutamate; however, intracellular threonine, lysine, and aspartate increased, while ornithine, alanine, glycine, and taurine decreased in co-stimulated cells from G-BCATm^KO^ mice compared to corresponding WT cells (Fig. 1e and Supplementary Table 6).

### Co-stimulated CD4^+^ T cells deficient in BCATm have increased mitochondrial respiration but reduced production of ATP via oxidative phosphorylation

Because BCATm is a mitochondrial enzyme previously associated with energy expenditure [22] we investigated the impact of a loss of BCATm on oxygen metabolism of CD4^+^ T cells. Unstimulated and TCR-stimulated cells showed small but significant increases in mitochondrial respiration and SRC (TCR-stimulated cells) in the absence of BCATm (Fig. 2a, b). The most prominent differences, however, were observed between co-stimulated CD4^+^ T cells. A loss of BCATm led to 1.04- and 0.9-fold increase in the mitochondrial respiration and SRC (Fig. 2a, b, co-stimulated cells). However, addition of oligomycin failed to reduce the OCR of BCATm-deficient CD4^+^ T cells to the levels observed in cells expressing BCATm (Fig. 2a, refer to co-stimulated cells after oligomycin addition). Subsequently, the mitochondrial ATP production was reduced by 64%, which correlated with a 69% drop in the coupling efficiency of the co-stimulated T cells from G-BCATm^KO^ mice (Fig. 2b). In contrast, these cells maintained very high glycolytic rate (a 3.1-fold increase) and associated glycolytic capacity (a 4.3-fold increase) compared to co-stimulated cells expressing BCATm (Fig. 2c, d).

Follow-up experiments were conducted using our newly created T-cell-conditional BCATm knockout mouse (T-BCATm^KO^), designed to explore the loss of BCATm in T cells under in vivo conditions (refer to Figs. 4–6). The loss of BCATm expression along with unaltered expression of BCATc and the BCKDH complex were verified in co-stimulated CD4^+^ T cells from T-BCATm^KO^ mice (Fig. 2e) followed by exploring the expression of Hexokinase II and the phosphorylation of the LDHA enzyme, which were found increased by 56% and 1.7-fold, respectively, suggesting that a loss of BCATm correlated with high glycolytic phenotype (Fig. 2f).

### Co-stimulated CD4^+^ T cells deficient in BCATm upregulate mTORC1 signalling, activate AMPK, and release more IFN-γ

Because we found elevated intracellular leucine but low mitochondrial ATP production in the BCATm-deficient co-stimulated CD4^+^ T cells (Figs. 1d and 2b), we next investigated the complex 1 of mTOR (mTORC1) and the energy sensor AMPK by measuring the phosphorylation states of the ribosomal protein S6 (a downstream target of mTORC1) and AMPK (Fig. 2g). While the 52% increase in P-S6 in the absence of BCATm indicated upregulation of mTORC1, there was a 2.8-fold increase in the phosphorylation and thus activation of AMPK (Fig. 2g). This suggested a compensatory mechanism to restore the energy balance upon loss of BCATm. Adding BCAT-IN-2 to co-stimulated CD4^+^ T cells of control T-BCATm^fl/fl^ mice to inhibit the enzyme activity of BCATm mimicked the effects of a loss of BCATm on hexokinase II, and the activation of LDHA, mTORC1 and AMPK suggesting the effects were dependent on catalytically active BCATm (Fig. 2f, g).

Lastly, we explored whether the high energy demand of the BCATm-deficient CD4^+^ T cells may lead to exhaustion. The marker of exhaustion TIM-3, but not LAG3, TCF1/7 or TIGIT, was elevated by ~30% in co-stimulated cells from T-BCATm^KO^ mice suggesting that a state of exhaustion was not established (Supplementary Fig. 2a). Moreover, when IFN-y levels were measured in the supernatants of these cells, IFN-y was increased by 7.2-, 1.4-, and 1-fold following 24 h, 48 h, and 72 h of activation compared to control T cells (Fig. 2h). Applying NALA to mimic leucine depletion, reversed these effects in T cells from T-BCATm^KO^ mice (Fig. 2h, 72 h only). Thus, in the absence of BCATm, co-stimulated CD4^+^ T cells were able to improve functionality. In contrast, the loss of BCATm had only mild effect on their ability to enter the cell cycle (Supplementary Fig. 2b, c). Taken together the results strongly indicate that BCATm exerts immunosuppressive pressure on T cells upon activation by negatively affecting their metabolic activity in mTORC1/AMPK dependent manner.

### The human BCAT2 gene shows a strong negative correlation with mitochondrial ribosome and ETC genes

**Fig. 3:**
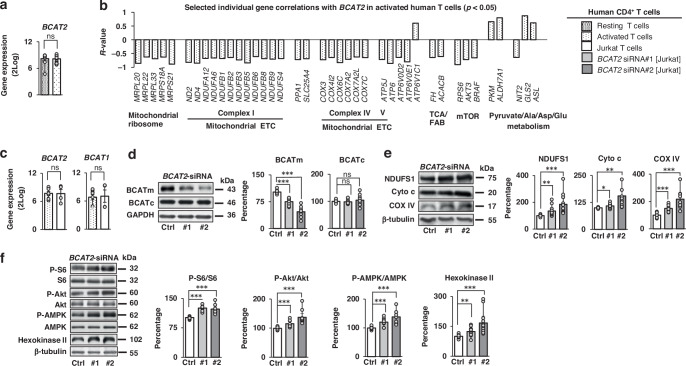
The human *BCAT2* gene shows a strong negative correlation with mitochondrial ribosome and electron transport chain (ETC) genes. **a** Comparison of the gene expression of *BCAT2* (the human gene for BCATm) between resting (*n* = 10) and activated (*n* = 11) CD4^+^ and CD8^+^ T cells from tonsils of healthy human donors. **b** Selected correlations of *BCAT2* with individual genes based on the KEGG pathway analysis of activated CD4^+^ and CD8^+^ T cells (*n* = 11) from tonsils of healthy human donors. **c** Comparison of the expression of *BCAT2* and *BCAT1* (the human gene for BCATc) between in vitro activated human CD4^+^ and CD8^+^ T cells (*n* = 8) and Jurkat T cells (*n* = 4). In **a**–**c** the gene expression profile of nonmalignant T cells and Jurkat T cells was deposited by Eckerle S and co-authors [18] (**a**, **b**) and Crescenzo R and co-authors (**c**) [19] in the R2: Genomics Analysis and Visualisation Platform (http://r2.amc.nl). **d**–**f** Human Jurkat T cells subjected to siRNA using 200 nM of Control-siRNA (Ctrl) or *BCAT2*-siRNA#1 and #2 for 72 h (*n* = 3 independent cultures/variant). The protein expression and the phosphorylation (P) states (S6, Akt, and AMPK only) of the following proteins were determined via western blotting: BCATm and BCATc (**d**) NDUFS1, cytochrome c (Cyto c) and COX IV (**e**) S6, Akt, AMPK, and Hexokinase II (**f**). Image J was used to quantify the relative band intensity of each protein as normalised to β-tubulin or GAPDH and presented as a percentage of control cells. Western blotting images were representative of 3 independent experiments. For all panels, average ± SEM. Statistical significance as determined by a two-tailed Student’s *t* test: *p ≤ 0.05, **p ≤ 0.01, ***p ≤ 0.001 or ns no significance.

Increased mitochondrial respiration of CD4^+^ T cells lacking BCATm cannot be explained by disrupted BCAA degradation. To address this, we accessed gene expression profiles of T cells from healthy human donors [18]. Comparison of the expression of the human *BCAT2* (encodes BCATm) in resting and activated T cells showed no significant differences between the two groups (Fig. 3a) consistent with the constitutive expression of *BCAT2*.

KEGG pathway analysis using *BCAT2* as the reporter gene in activated human T cells revealed 351 statistically significant combinations for *BCAT2* and genes from different KEGG pathways (Supplementary Table 2, Fig. 3b). The first five KEGG pathways included 31.9% of the significant gene correlations with *BCAT2*. The ribosome and oxidative phosphorylation KEGG pathways were the 2nd and the 3rd most significant pathways, respectively. The correlations between genes encoding for the cytosolic and mitochondrial ribosomes, complex I, IV and V of ETC and *BCAT2* were entirely negative (Fig. 3b, Supplementary Tables 2–3). In addition, 2 genes from the TCA cycle and fatty acid metabolism and 3 genes from the mTOR pathway were found in a strong negative correlation with *BCAT2*, while no correlations were found with genes encoding uncoupling proteins or AMPK (Fig. 3b and Supplementary Table 3). This previously unknown correlation between *BCAT2* and ribosome/mitochondrial ETC genes suggested a negative regulatory role of BCATm that may go beyond its catalytic activity.

To verify these findings on protein level, we used the Jurkat T cells as an experimental model. Comparison of leucine metabolism in Jurkat T cells and mouse WT T cells, confirmed that Jurkat T cells committed most of leucine metabolism (96%) to leucine transamination (Supplementary Fig. 3a, b). Given the T-ALL origin of Jurkat T cells, we compared the expression of *BCAT2* and *BCAT1* between T cells from healthy human donors and patients with T-ALL (Fig. 3c). Finding no difference, we next subjected the Jurkat T cells to siRNAs targeting *BCAT2*. *BCAT2*-siRNA#1 caused ~27% and *BCAT2*-siRNA#2 caused ~54% reduction in the protein expression of BCATm while that of BCATc was unaffected (Fig. 3d). The two knockdown variants of *BCAT2* caused significant increases in the protein expression of components of ETC, such as NDUFS1 (35% [#1] and 85% [#2]), cytochrome c (9% [#1] and 55% [#2]) and COX IV (49% [#1] and 2.1-fold [#2]) (Fig. 3e). Furthermore, we recorded significant increases in the activation of mTORC1 (P-S6 increased by 25% [#1] and 23% [#2]), mTORC2 (P-Akt increased by 16% [#1] and 38% [#2]) and AMPK (P-AMPK increased by 21% [#1] and 39% [#2]) (Fig. 3f). Hexokinase II was also upregulated by 24% [#1] and 67% [#2] (Fig. 3f). The effects were reminiscent of these obtained with co-stimulated mouse CD4^+^ T cells deficient in BCATm but lower due to the partial loss of BCATm from Jurkat T cells. A knockdown of BCATm did not cause biologically significant changes in the viability or proliferation of the Jurkat T cells (Supplementary Fig. 3c–e).

### BCATm-deficient CD8^+^ T cells upregulate mTORC1 signalling and secrete more granzyme B and perforin during expansion

**Fig. 4:**
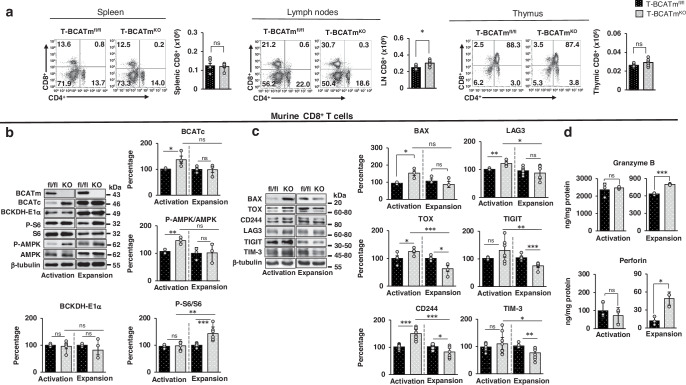
A loss of BCATm in murine CD8^+^ T cells leads to increased mTORC1 signalling and decreased T cell exhaustion during their expansion phase **a** Spleens, lymph nodes and thymuses from naïve T-BCATm^fl/fl^ (fl/fl) and T-BCATm^KO^ (KO) mice were used to quantify total CD4^+^ and CD8^+^ T cells. Representative density plots of the co-expression of CD4 and CD8 surface markers are shown, along with average data ± SEM of mixed sex, *n* = 6 mice/genotype. **b**–**d** CD8^+^ T cells from T-BCATm^fl/fl^ and T-BCATm^KO^ mice were activated for 48 h (activation phase) and expanded to 96 h (expansion phase) as detailed in the Methods. In **b**, **c** protein expression of BCATm, BCATc, BCKDH-E1α, S6, and AMPK and phosphorylation (P-S6, P-AMPK) states, BAX, TOX, CD244, LAG3, TIGIT, and TIM-3 were assessed by western blotting. Image J was used to quantify the relative band intensity of these proteins as normalised to β-tubulin or the ratio between P-S6 and S6 and P-AMPK and AMPK, respectively, and presented as percentage of floxed control. The western blotting images were representative of 3 or more independent experiments. In **d** ELISA on supernatants from CD8^+^ T cells to determine the secretion of granzyme B and perforin. In **b**–**d** average ± SEM of *n* = 6–9 mice/genotype, mixed sex. Statistical significance as determined by a two-tailed Student’s *t* test: *p ≤ 0.05, **p ≤ 0.01, ***p ≤ 0.001 or ns no significance.

Before exploring the role of BCATm in anti-lymphoma T-cell immunity, we investigated whether BCATm affects CD4^+^ and CD8^+^ T cells in the naïve animal. Splenic and thymic CD4^+^ and CD8^+^ T cell populations did not differ between T-BCATm^fl/fl^ and T-BCATm^KO^ mice; however, there was a small significant increase in CD8^+^ T cells in lymph nodes of T-BCATm^KO^ mice (Fig. 4a, average values for CD4^+^ T cells not shown).

To understand the impact of BCATm on CD8^+^ T-cell functionality, we looked at the activation and expansion of these cells since they experience distinct metabolic shifts when transitioning between the two phases [26, 27]. Western blotting of CD8^+^ T cells confirmed no expression of BCATm or changes in BCKDH-E1α in either phase (Fig. 4b). However, during the activation phase, the loss of BCATm from CD8^+^ T cells correlated with significant increases in the expression of BCATc (36%), the activation of AMPK (36%), the expression BAX (61%), or the exhaustion markers TOX (25%), CD244 (47%), and LAG3 (21%) when compared to CD8^+^ T cells expressing BCATm (Fig. 4b, c). No changes were recorded for granzyme B or perforin (Fig. 4d, activation). These results suggested that the loss of BCATm contributed to a catabolic state during the activation phase. In contrast, the loss of BCATm during the expansion phase correlated with upregulation of mTORC1 (P-S6 increased by 43%). As the CD8^+^ T cells deficient in BCATm transitioned to expansion, they showed significant reductions in TOX [50%], CD244 [45%], LAG3 [29%], TIGIT [43%], and TIM-3 [30%] (Fig. 4c, compare the grey bars). These cells released 26% and 3.1-fold more granzyme B and perforin, respectively (Fig. 4d, expansion). Thus, a loss of BCATm from CD8^+^ T cells may help maintain an anabolic and fully functional state during the phase of expansion.

### A single deletion of BCATm from T cells leads to local but not systemic resistance to EL4-OVA lymphoma

**Fig. 5:**
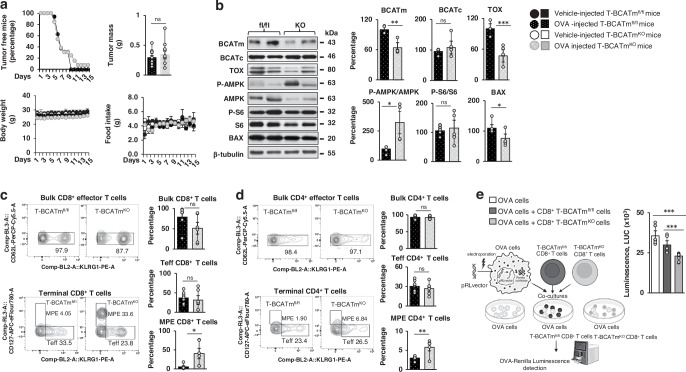
A single loss of BCATm in murine T cells affords local but not systemic resistance to EL4-OVA lymphoma. **a**, **b** Male T-BCATm^fl/fl^ (fl/fl) and T-BCATm^KO^ (KO) mice that were vehicle (*n* = 3 mice/genotype) or tumour (*n* = 9–12 mice /genotype) injected were monitored for 15 days. In **a**, tumour free mice, tumour mass, body weight and food intake. In **b** western blotting of tumour tissues showing the expression of BCATm, BCATc, TOX, BAX, AMPK, and S6 along with the phosphorylation (P-AMPK, P-S6) states. Image J was used to quantify the relative band intensity of BCATm, BCATc, TOX, and BAX as normalised to β-tubulin, or the ratio between P-AMPK and AMPK or P-S6 and S6, respectively, and presented as percentage of floxed control. The western blot images were representative of 6–9 tumour lysates/genotype. In **c**, **d** EL4-OVA tumours from T-BCATm^fl/fl^ and T-BCATm^KO^ mice were collected at day 10, homogenised into cell suspensions and stained for CD4, CD8, CD62L and KLRG1 to quantify the percentage of bulk effector, terminal effector (Teff), and memory precursor effector (MPE) CD4^+^ and CD8^+^ T cells, respectively. Representative flow density plots are shown, along with average data, *n* = 6 mice/genotype. In **e** simplified schematics of the steps of the cytotoxic killing assay showing EL4-OVA cells transfected with pRenilla and co-cultured with expanded CD8^+^ T cells from spleens of T-BCATm^fl/fl^ or T-BCATm^KO^ mice, *n* = 5 mouse spleens (mixed sex) per variant. Luminescence (LUC) generated by the renilla luciferase enzyme carried by the EL4-OVA cells. For all panels, average ± SEM of mixed sex. Statistical significance as determined by a two-tailed Student’s *t* test or One-way Anova: *p ≤ 0.05, **p ≤ 0.01, ***p ≤ 0.001 or ns no significance.

Prompted by the impact of a loss of BCATm on CD4^+^ and CD8^+^ T cells in vitro, we sought to understand whether T-BCATm^KO^ mice can overcome OVA-expressing-EL4 lymphoma (Supplementary Fig. 4a–c and Fig. 5a). All T-BCATm^fl/fl^ mice were tumour-bearing by day 10, while 25% of T-BCATm^KO^ mice remained tumour-free. On day 15, 8% of T-BCATm^KO^ mice did not have palpable tumours; however, we found no difference in tumour masses between T-BCATm^fl/fl^ and T-BCATm^KO^ mice (Fig. 5a). Similar results were obtained with female mice (Supplementary Fig. 4c). All mice tolerated the lymphoma challenge well as seen by body weight, food intake or organ weights (Fig. 5a, Supplementary Fig. 4c, Supplementary Tables 7–8).

Tumours from T-BCATm^KO^ mice revealed significantly reduced expressions of BCATm [36%] and TOX [53%], while the activity of AMPK [a 2.5-fold increase], but not mTORC1, was upregulated (Fig. 5b). These molecular changes were not sufficient to cause reduction in tumour growth. Tumoral populations of bulk, terminal and memory precursor effector CD4^+^ and CD8^+^ T cells, however, revealed a 1.02- and 6.8-fold increases in the memory precursor CD4^+^ and CD8^+^ T cells, respectively, in tumours from T-BCATm^KO^ mice (Fig. 5c, d).

Because of the changes in the molecular signature of the OVA-tumours and the tumoral memory precursor CD8^+^ T cells in the T-BCATm^KO^ mice, we assessed whether BCATm-deficient CD8^+^ T cells could directly destroy EL4-OVA cells. Using an in vitro cytotoxic assay, EL4-OVA cells, expressing the Renilla luciferase, were co-cultured with CD8^+^ T cells from T-BCATm^fl/fl^ or T-BCATm^KO^ mice. CD8^+^ T cells from T-BCATm^fl/fl^ and T-BCATm^KO^ mice caused 16% and 37% reductions in EL4-OVA luminescence, respectively, demonstrating that the CD8^+^ T cells from T-BCATm^KO^ mice had higher cytotoxic ability (Fig. 5e).

### BCATm contributes to reduction in EL4-OVA tumour burden in a double combined deletion with BCATc

**Fig. 6:**
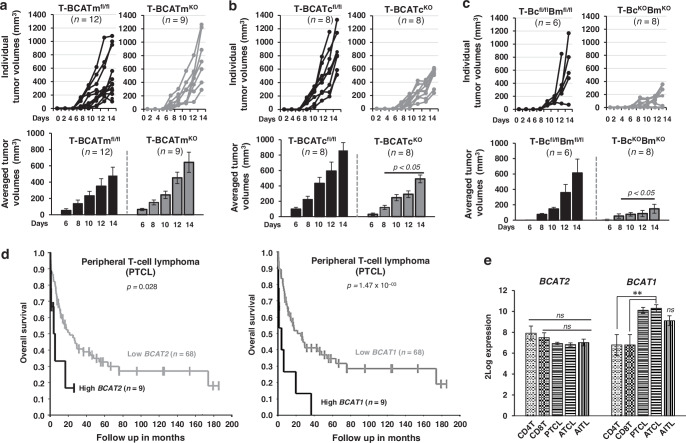
A combined BCATc-BCATm deletion from T cells significantly improves mice ability to fight lymphoma tumours. In **a–c** Comparison of individual and average tumour volumes over time following s.c. inoculation with 2.5×10^5^ EL4-OVA cells of the single T-cell-conditional BCATm (T-BCATm^KO^) (**a**), the single T-cell-conditional BCATc (T-BCATc^KO^) (**b**), and the double T-cell-conditional BCATc-BCATm (T-Bc^KO^Bm^KO^) (**c**) knockout mouse models. Tumours appeared around days 5–6 and were measured until day 14 before mouse sacrifice. Each knockout model (grey lines) was first compared to corresponding floxed control (black lines) and their percentage difference was used to compare tumour growth between the different mouse models. The number of mice/group is indicated in each graph. In the average graphs, data are ±SEM of male sex, age between 8 and 12 weeks old. Statistical significance as determined by a two-tailed Student’s *t* test: *p* ≤ 0.05 as compared to the corresponding control as indicated in the graphs. In **d** Kaplan–Meier curve comparing the overall survival probability of patients diagnosed with peripheral T cell lymphoma (PTCL, *n* = 193) and expressing low and high *BCAT2* (the human gene encoding BCATm) and *BCAT1* (the human gene encoding BCATc). In **e** comparison of *BCAT1* and *BCAT2* expression between CD4^+^, CD8^+^ T cells from healthy human donors (*n* = 4/T cell type), and lymphoma biopsies categorised as follows: peripheral T-cell lymphoma (PTCL, *n* = 35), anaplastic T-cell lymphoma (ATCL, *n* = 23), and angioimmunoblastic T-cell lymphoma (AITL, *n* = 24). The gene expression was measured in 2 log. Statistical significance as determined by a two-tailed Student’s *t* test or One-way Anova: *p ≤ 0.05, **p ≤ 0.01, ***p ≤ 0.001 or ns no significance.

Considering that a single loss of BCATm from T cells was not sufficient to induce reduction in EL4-OVA growth, we asked if a combined loss of BCATc and BCATm from mouse T cells may produce such effect. We used three mouse models (T-BCATm^KO^, T-BCATc^KO^, and T-Bc^KO^Bm^KO^) and littermate controls (T-BCATm^fl/fl^, T-BCATc^fl/fl^, T-Bc^fl/fl^Bm^fl/fl^). The mouse with a double BCATc-BCATm deletion from T cells (T-Bc^KO^Bm^KO^ mice) was characterised as shown in Supplementary Fig. 5, while the T-BCATc^KO^ mouse was only used as an additional control. The individual and average/day tumour growth for KO mice (grey lines) relative to littermate controls (black lines) showed no difference for mice carrying a single BCATm deletion in T cells (Fig. 6a). However, the EL4-OVA tumour growth was reduced on average between 43% and 51% in T-BCATc^KO^ mice and between 68% and 76% in T-Bc^KO^Bm^KO^ mice (Fig. 6 b, c, days 10–14). Therfore, the loss of BCATm from T cells may have accounted for an additional 20–25% reduction in tumour growth by the end of the study contributing to the anti-lymphoma T-cells immunity.

To understand the clinical relevance of the BCAT isoenzymes, we analysed specimens from PTCL patients revealing that patients with high expression of *BCAT2* or *BCAT1* had significantly worse overall survival (Fig. 6d). Furthermore, both isoenzymes were expressed in different subtypes of T-cell lymphomas. *BCAT1*, however, was significantly overexpressed in PTCL and ATCL compared to T cells from healthy donors (Fig. 6e). Taken together with the in vivo EL4-OVA studies, targeting *BCAT1* may be more suitable for a single therapy, while *BCAT2* for combinatorial therapies.

## Discussion

T cells entering the TME face metabolic barriers that restrain their effector function. Among those barriers, depletion of essential amino acids, including leucine, contributes to T-cell dysfunction [1, 28]. Being responsible for the mitochondrial transamination of leucine, BCATm may constitute a barrier to its continuous supply for T cells. This is partially supported by transcriptomics, metabolomics, or multi-omics reports; however, such studies provided little to no experimental evidence [14, 29]. In lieu of recent discoveries on T-cell mitochondria in the interactions between cancer and immune cells [4, 17], it is important to focus scientific attention to mitochondrial proteins, such as BCATm, that may have drug targetable potential.

This report demonstrated that BCATm is a part of the basic metabolic machinery of T-cell mitochondria; however, BCATm continued to transaminate BCAAs upon T-cell activation despite induction of BCATc. Deleting BCATm from CD4^+^ T cells eliminated the mitochondrial step of BCAA degradation and while this elevated intracellular BCAAs and increased mitochondrial respiration, co-stimulated cells were unable to maintain mitochondrial ATP production due to reduced coupling efficiency. Because this state of high energy demand could not be met by basal respiration, the cells adopted a highly glycolytic phenotype. These effects were reproduced in control cells by applying BCAT-IN-2 implying that disruption in the enzymatic function of BCATm was likely responsible for the increased mitochondrial energy demand. In contrast, maximal respiration correlated with an increased ATP production in lymphoma cells treated with increasing leucine concentrations [15].

The regulation of the nutrient sensing pathway that involves the opposing kinases AMPK and mTOR is well established to depend on leucine. As shown in rat muscle cells, leucine mediates protein synthesis by decreasing the activation of AMPK while upregulating mTORC1 [30]. Moreover, the AMPK-mTOR signalling axis was affected by valine-dependent upregulation of BCATm in bovine mammary cells, which led to increases in TCA cycle intermediates, inhibition of AMPK and activation of mTORC1 [31]. Despite the increased leucine concentrations, such regulation was not observed in CD4^+^ T cells deficient in BCATm in this report. Some clues may come from the earlier characterisation of the G-BCATm^KO^ mouse [22]. This mouse adopted a unique phenotype characterised by highly elevated plasma BCAAs, low body fat, and increased oxygen consumption and energy expenditure in brown fat and skeletal muscles, which were linked to protein turnover futile cycle. The transcriptomic analysis in the current study further revealed that the human *BCAT2* gene for BCATm was in negative correlation with genes encoding cytosolic and mitochondrial ribosomes and components of ETC suggesting a novel regulatory role of BCATm in protein translation/mitochondria of T cells that may go beyond its catalytic function. Pyruvate kinase M2 exists in transcriptionally active dimeric (di-PKM2) or enzymatically active tetrameric form (tet-PKM2) demonstrating gene and metabolic effects [32]. Activation of this enzyme reduces exhaustion, increases mitochondrial reorganisation, and enhances the metabolic fitness of T cells [32].

While the canonical interplay between mTOR and AMPK was not observed in the CD4^+^ T cells, the adoption of a glycolytic phenotype in the absence of BCATm was dependent on the mTOR-AMPK axis. A mechanistic detail of this unique phenotype may involve activation of mTORC1 by the elevated BCAAs, but also adjustment of the energy status via concurrent activation of AMPK as means of adaptation. In other studies, AMPK was shown to directly activate mTOR2 under acute energetic stress as a mechanism to promote tumorigenesis despite AMPK’s well-known tumour-suppressive functions [33]. Likewise, in response to amino acid re-addition, C2C12 myocytes were able to activate concurrently AMPK and mTOR [34].

CD8^+^ T cells from the T-BCATm^KO^ mice showed a distinctive shift in the AMPK-mTOR axis, where AMPK, but not mTORC1 activity, was upregulated during their activation followed by upregulation of mTORC1, but suppression of AMPK activity, during their expansion. Similarly, the exhaustion markers TOX, LAG-3, and CD244, which are key in defining dysfunctional CD8^+^ T cells under chronic conditions including lymphocytic malignancies [35–38]. were upregulated in the absence of BCATm during activation but as these cells transitioned to expansion all exhaustion markers, including TIGIT and TIM-3, were significantly downregulated and the levels of secreted granzyme B and perforin increased. These findings suggested that BCATm may play a regulatory role in the establishment of an anabolic state improving the functionality of CD8^+^ T cells.

While BCATc has been explored in immune cells [21, 39–41], the role of BCATm is only emerging. A *Bcat2* knockdown in bone marrow-derived macrophages from wild-type mice suppressed M2 polarisation [42]. In another study, there was a reverse relationship between the mRNA expression of *Bcat2* and the percentage of circulating IL-17^+^CD4^+^ T cells or neutrophils in the peripheral blood or lymph nodes of a mouse model of psoriasis [43]. Taken together with our report, these studies implied that BCATm may have an immunosuppressive function.

Contrary to immune cells, BCATm has been explored in cancer cells [2]. BCATm is overexpressed in most but not all cancer types [10, 44, 45]. According to Mayers and co-authors, cancers with different tissue-of-origin have different preferences for BCAAs and BCAA metabolism. NSCLC utilises a large amount of BCAAs as a nitrogen source for biosynthesis. Subsequently, NSCLC tumours are impaired when deficient in BCATm. In contrast, pancreatic ductal adenocarcinoma (PDAC) has decreased BCAA uptake and does not require upregulation of BCATm [9]. However, deletion of the mitochondrial malic enzyme M3 in PDAC caused upregulation of BCATm in AMPK-dependent manner leading to increased mitochondrial BCAA transamination and regeneration of glutamate for nucleotide biosynthesis [46]. This is explained by glutamate supporting the glutamine-arginine axis, an important giveaway for cancer growth [47]. Interestingly, we found glutamine and arginine elevated in CD4^+^ T cells deficient in BCATm. While we previously found low glutamate and glutamine in plasma and tumour tissues of G-BCATm^KO^ mice [15] such reduction could not be observed in the current report, since the cells were grown in medium enriched in glutamine. We, however, observed an increased uptake of glutamine and higher intracellular glutamine in co-stimulated CD4^+ ^T cells deficient in BCATm. An explanation of this finding may be that leucine and glutamine concentrations depend on the obligate exchanger SLC7A5/SLC3A2, which uses intracellular glutamine as an efflux substrate to regulate the uptake of extracellular leucine [48, 49]. Since the deletion of BCATm resulted in increased intercellular leucine, this may have reduced the necessity for the exchange with glutamine.

While we demonstrated that CD8^+^ T cells had enhanced ability to combat EL4-OVA lymphoma cells in the absence of BCATm, the T-BCATm^KO^ mice failed to respond to the in vivo lymphoma challenge. This contrasted with our earlier study using the G-BCATm^KO^ mice and different BCAA diet formulations. The G-BCATm^KO^ mice had delayed lymphoma growth, which correlated with significant increases in plasma BCAAs when fed normal BCAA diet [15]. The immune profile of the EL4-OVA tumours isolated from the T-BCATm^KO^ mice revealed increased populations of CD4^+^ and CD8^+^memory precursor T cells, which cells correlate with better outcomes in cancer patients [50, 51]. Corresponding reduction in EL4-OVA growth was observed, however, only when BCATm was deleted from mouse T cells in a combination with BCATc. In bladder cancer a negative correlation was found between the secretion of cytokines from cytotoxic T cells and BCATm^+^ tumour cells. Moreover, when BCATm was downregulated in bladder cancer the efficacy of anti-PD1 therapy was enhanced [13]. The applicability of such studies in clinical trials, however, needs to be carefully evaluated given that not all cancers overexpress BCATm [45]. Lastly, while our objective was to evaluate the role BCATm plays in T cells, exploring the effect of BCAA diets on T-cell conditional mouse models may prove informative in future clinical applications.

In summary, this study undertook a comprehensive approach in characterising BCATm in T cells using several mouse models and human T cells. The findings revealed a previously unknown role of BCATm deficiency in T-cell mitochondria that triggered a compensatory mechanism in mTOR-AMPK dependent manner that ultimately afforded the T cells with better functionality including improved ability of the CD8^+^ T cells to combat EL4-OVA cells. Thus, BCATm may play an immunosuppressive role in the context of the T-cell-driven immunity against lymphoma, however, the effects of BCATc appeared superior judged by the ability of BCATc-deficient T cells to reduce EL4-OVA tumour burden alone. While a single loss of BCATm was unsuccessful in providing a systemic response, the results obtained with our double T cell-conditional knockout mouse suggested that BCATm plays a supportive role. Thus, BCATm may have the potential to be targeted for local anti-tumour immunity, alone, or in combinatorial therapies.

## Supplementary information

Supplementary Tables 1-8

Supplementary Figures 1-5

Supplementary Information

## Data Availability

This manuscript did not generate datasets; however, the authors will consider investigators’ requests for access to data supporting the results reported in the manuscript.
