# Supplementary material for: Blocking mitochondrial leucine transamination enhances T-cell activation and improves T-cell immunity against OVA-producing EL4 lymphoma
Source: Br J Cancer. 2026 May 5;135(3):406–17. doi: 10.1038/s41416-026-03455-5 (PMC13372810; doi:10.1038/s41416-026-03455-5)
Supplement: Supplementary file 1 — Supplementary Tables 1-8 [file 41416_2026_3455_MOESM1_ESM.docx]

| **Supplementary Table 1.** List of antibodies used in the manuscript | | | |
| --- | --- | --- | --- |
| **Antibody name** | **Company and Cat #** | **Name and function of protein of interest*** | **Protein size (kDa)** |
| AMPKα (23A3) rabbit mAb | *Cell Signaling Tech*  *Cat # 2603* | Α subunit of AMP-activated protein kinase (AMPK) playing a key role in the regulation of energy homeostasis. | 62 |
| Phospho-AMPKα (Thr172) (40H9) rabbit mAb | *Cell Signaling Tech*  *Cat # 2535* | *Endogenous AMPKα1 phosphorylated at threonine 183 and endogenous AMPKα2 phosphorylated at threonine 172. Phosphorylation is required for AMPK activation.* | 62 |
| Akt pAb | *Cell Signaling Tech*  *Cat # 9272* | *v-akt murine thymoma viral oncogene homolog (Akt) plays a role in cell growth by directly phosphorylating mTOR in a rapamycin-sensitive complex containing raptor.* | 60 |
| Phospho-Akt (Ser473) (193H12) mAb | *Cell Signaling Tech*  *Cat # 4058* | *Phosphorylation is required for Akt activation.* | 60 |
| Bax (D3R2M) rabbit mAb | *Cell Signaling Tech*  *Cat #14796* | *Bcl-2-associated X protein (Bax) is a key component for cellular induced apoptosis through mitochondrial stress.* | 20 |
| BCATc rabbit mAb | *In house (ref. 21)* | *Branched-chain aminotransferase isoenzyme responsible for the cytosolic transamination of BCAAs.* | 46 |
| BCATm rabbit mAb | *In house (ref. 21)* | *Branched-chain aminotransferase isoenzyme responsible for the mitochondrial transamination of BCAAs.* | 43 |
| BCKDH-E1α rabbit pAb | *In house (ref. 21)* | *E1α subunit of the branched-chain α-keto acid dehydrogenase involved in the second step of BCAA catabolism.* | 49 |
| BCKDH-E2 rabbit pAb | *In house (ref. 21)* | *E2 is dihydrolipoamide branched-chain transacylase involved in the second step of BCAA catabolism.* | 53 |
| β-tubulin (9F3) rabbit mAb | *Cell Signaling Tech*  *Cat # 2128* | *β-tubulin was used as a loading control.* | 55 |
| CD244 rabbit mAb | *Cell Signaling Tech*  *Cat # 54560* | *The 2B4/SLAMF4/CD244 (D5J9D) antibody detects endogenous levels of the CD244 protein. T cell exhaustion relates to an increase in the expression of the CD244 receptor on immune cells, particularly T cells and NK cells.* | 70-120 |
| CD62L-PerCP-Cy5.5 rat mAb  Rat IgG2a kappa – PerCP-Cy5.5 | *Thermo Scientific*  *Cat # 45-0621-82*  *Thermo Scientific*  *Cat # 45-4321-80* | *For detection of central memory CD4^+^ and CD8^+^ T cells.*  *Isotope control for CD62L during Flow cytometry.* | n/a |
| CD127-APC-eFuor780 rat mAb  Rat IgG2a kappa – APC-eFluor780 | *Thermo Scientific*  *Cat # 47-1271-82*  *Thermo Scientific*  *Cat # 47-4321-82* | *For detection of memory precursor CD4^+^ and CD8^+^ T cells.*  *Isotope control for CD127 during Flow cytometry.* | n/a |
| CD4-Alexafluor488 rat mAb  Rat IgG2b kappa-Alexafluor488 mAb | *Thermo Scientific*  *Cat # 53-0041-82*  *Thermo Scientific*  *Cat # 53-4031-80* | *For detection of CD4^+^ expressing T cells via Flow cytometry.*  *Isotope control for CD4^+^ T cells during Flow cytometry.* | n/a |
| CD8-PE-Cy7 rat mAb  Rat IgG2a kappa-PE-Cy7 mAb | *Thermo Scientific*  *Cat # A15385*  *Thermo Scientific*  *Cat # 25-4321-82* | *For detection of CD8^+^ expressing T cells via Flow cytometry.*  *Isotope control for CD8^+^ T cells during Flow cytometry.* | n/a |
| Cytochrome c rabbit mAb | *Cell Signaling Tech*  *Cat # 11940* | *Cytochrome c is an electron-transport protein part of the respiratory chain localized to the mitochondrial intermembrane space.* | 20 |
| COX IV (3E11) rabbit mAb | *Cell Signaling Tech*  *Cat # 4850* | *Cytochrome c oxidase (COX) is a hetero-oligomeric enzyme consisting of 13 subunits localized to the inner mitochondrial membrane.* | 17 |
| Hexokinase II (C64G5) rabbit mAb | *Cell Signaling Tech*  *Cat # 2867* | *Hexokinases II is associated with the outer mitochondrial membrane and is critical for maintaining an elevated rate of aerobic glycolysis in cancer cells (Warburg Effect).* | 102 |
| Horseradish peroxidase-conjugated AffiniPure donkey anti-rabbit IgG | *Jackson ImmunoResearch*  *Cat# 711-035-152* | Used as the secondary antibody during western blotting. | n/a |
| KLRG1 mAb (2F1), PE  Syrian Hamster IgG Isotype Control, PE, | *Thermo Scientific*  *Cat # 12-5893-82*  *Thermo Scientific*  *Cat # 12-4914-81* | *Killer cell lectin-like receptor G1 (KLRG1) was used for detection of terminal effector CD4^+^ and CD8^+^ T cells.*  *Isotope control for KLRG1 during Flow cytometry.* | n/a |
| LAG3 (E5S8V) rabbit mAb | *Cell Signaling Tech*  *Cat # 80282* | *Lymphocyte activation gene 3 (LAG3, CD223) is an immune checkpoint control protein that negatively regulates T cells.* | 60-80 |
| LDHA/LDHC (C28H7) rabbit mAb | *Cell Signaling Tech*  *Cat # 3558* | *Lactate dehydrogenase (LDH) catalyzes the interconversion of pyruvate and NADH to lactate and NAD+.* | 37 |
| Phospho-LDHA (Tyr10) rabbit mAB | *Cell Signaling Tech*  *Cat # 8176* | *Phosphorylation at Tyr10 up-regulates the activity of LDHA and is found in a variety of human cancer cells.* | 37 |
| NDUFS1 (E4K3E) rabbit mAb | *Cell Signaling Tech*  *Cat # 70264* | *NADH dehydrogenase Fe-S protein 1 (NDUFS1) is a nuclear encoded structural subunit of NADH: ubiquinone oxidoreductase (complex 1) in the mitochondrial electron transport chain.* | 75 |
| Rb(D20) rabbit mAb | *Cell Signaling Tech*  *Cat # 9313* | *Retinoblastoma (Rb) controls progression through the restriction point within the G1-phase of the cell cycle.* | 110 |
| Phospho-Rb(Ser807/811)(D20B12) rabbit mAb | *Cell Signaling Tech*  *Cat # 8516* | *Phosphorylation of Rb by the CDK4/6 cyclin D complex inhibits Rb binding to E2F allowing cell cycle progression.* | 110 |
| S6 ribosomal protein (5G10) rabbit mAb | *Cell Signaling Tech*  *Cat # 2217* | *S6 ribosomal protein, a downstream target of complex 1 of mTOR pathway.* | 32 |
| Phospho-S6 ribosomal protein (Ser240/244)(D68F8) rabbit mAb | *Cell Signaling Tech*  *Cat # 5364* | *Phosphorylation of S6 ribosomal protein correlates with an increase in translation of mRNA transcripts that contain an oligopyrimidine tract in their 5' untranslated regions.* | 32 |
| TCF1/TCF7 (C63D9) rabbit mAb | *Cell Signaling Tech*  *Cat # 2203* | *T-cell-specific transcription factor 1/7 (TCF1/TCF7) display dynamic expression in the total amount and the type of isoforms expressed in T cells during development and differentiation.* | *48-50* |
| TIGIT (E6Q3G) rabbit mAb | *Cell Signaling Tech*  *Cat # 25265* | *T-cell immunoreceptor with Ig and ITIM domains (TIGIT), also known as VSIG9, VSTM3, and WUCAM, is a member of the poliovirus receptor family of immunoglobulin proteins.* | *14, 30-50* |
| TIM-3 (D3M9R) rabbit mAb | *Cell Signaling Tech*  *Cat # 83882* | *TIM-3 is an inhibitory molecule that is induced following T-cell activation. TIM-3 is expressed by exhausted T cells in the settings of chronic infection and cancer.* | *45-80* |
| Tox/Tox2(E6G50) rabbit mAb | *Cell Signaling Tech*  *Cat # 36778* | *Thymocyte selection-associated high mobility group box protein (Tox) is a DNA-binding nuclear factor commonly induced by high antigen stimulation during chronic viral infection or cancer.* | *60-80* |
| *Information was taken verbatim from the manufacturer’s website  Antibodies dilutions were experimentally adjusted for each protein of interest and biological material tested.  mAb, monoclonal antibody  pAb, polyclonal antibody  n/a, not applicable | | | |

| **Supplementary Table 2.** KEGG pathway analysis showing the top five statistically significant KEGG pathways for the human *BCAT2* gene in activated human T cells | | | | |
| --- | --- | --- | --- | --- |
| **KEGG pathway** | **Set of 351 significant combinations** | **Total of 4040 combinations** | **Percentage of total** | ***P* value** |
|  | n of genes in set | n of genes in set | % | <0.05 |
| **Parkinson’s disease** | 27 | 108 | 25.0 | 1.76x10^-9^ |
| **Ribosome** | 25 | 102 | 24.5 | 1.40x10^-8^ |
| **Oxidative phosphorylation** | 24 | 105 | 22.9 | 2.54x10^-7^ |
| **Huntington’s disease** | 30 | 152 | 19.7 | 1.32x10^-6^ |
| **Glycine, serine, threonine metabolism** | 6 | 22 | 27.3 | 1.97x10^-3^ |
| Source, R2: Genomics Analysis and Visualization Platform (<http://r2.amc.nl>). The genome samples used in this analysis are from activated CD4^+^ and CD8^+^ T cells isolated from tonsils of healthy human donors (n=11) and deposited in R2 by Eckerle and co-authors (ref. 18). Ony the first five KEGG pathways are shown. | | | | |

| **Supplementary Table 3.** KEGG pathway analysis of activated T cells from human tonsils (n=11) | | | | |
| --- | --- | --- | --- | --- |
| ***Pathways and genes significantly correlated with BCAT2* (the human gene for BCATm)** | | | | |
|  |  | **Negative Correlations** |  |  |
|  | **Gene** | **Gene description** | **R-value** | **P-value** |
| ***Cytosolic ribosome*** | *RPL12*  *RPL14*  *RPL22*  *RPL24*  *RPL31*  *RPL34*  *RPL35A*  *RPL38*  *RPL6*  *RPL7*  *RPL9*  *RPS17*  *RPS21*  *RPS23*  *RPS27L*  *RPS28*  *RPS29*  *RPS4X*  *RPS6*  *RSL24D1* | *Ribosomal protein L12*  *Ribosomal protein L14*  *Ribosomal protein L22*  *Ribosomal protein L24*  *Ribosomal protein L31*  *Ribosomal protein L34*  *Ribosomal protein L35A*  *Ribosomal protein L38*  *Ribosomal protein L6*  *Ribosomal protein L7*  *Ribosomal protein L9*  *Ribosomal protein S17*  *Ribosomal protein S21*  *Ribosomal protein S23*  *Ribosomal protein S27L*  *Ribosomal protein S28*  *Ribosomal protein S29*  *Ribosomal protein S4X*  *Ribosomal protein S6*  *Ribosomal protein L24 domain containing 1* | -0.727  -0.604  -0.641  -0.763  -0.728  -0.938  -0.802  -0.764  -0.690  -0.741  -0.832  -0.620  -0.705  -0.865  -0.794  -0.682  -0.645  -0.671  -0.903  -0.708 | 0.01  0.05  0.03  0.006  0.01  0.00002  0.003  0.006  0.02  0.01  0.001  0.04  0.01  0.0006  0.004  0.02  0.03  0.02  0.0001  0.02 |
| ***Mitochondrial ribosome*** | *MRPL20*  *MRPL22*  *MRPL33*  *MRPS18A*  *MRPS21* | *Mitochondrial ribosomal protein L20*  *Mitochondrial ribosomal protein L22*  *Mitochondrial ribosomal protein L33*  *Mitochondrial ribosomal protein S18A*  *Mitochondrial ribosomal protein S21* | -0.856  -0.626  -0.688  -0.630  -0.885 | 0.0008  0.04  0.02  0.04  0.0003 |
| ***Oxidative phosphorylation*** |  |  |  |  |
| ***ETC Complex I*** | *ND2*  *ND4*  *NDUFA12*  *NDUFA6*  *NDUFB1*  *NDUFB2*  *NDUFB3*  *NDUFB5*  *NDUFB6*  *NDUFB8*  *NDUFB9*  *NDUFS4* | *MTND2* *Mitochondrially Encoded NADH: Ubiquinone Oxidoreductase Core Subunit 2*  *NADH dehydrogenase, subunit 4 (complex I)*  *NADH dehydrogenase (ubiquinone) 1 alpha subcomplex, 1*  *NADH dehydrogenase (ubiquinone) 1 alpha subcomplex, 6, 14kDa*  *NADH dehydrogenase (ubiquinone) 1 beta subcomplex, 1, 7kDa*  *NADH dehydrogenase (ubiquinone) 1 beta subcomplex, 2, 8kDa*  *NADH dehydrogenase (ubiquinone) 1 beta subcomplex, 3, 12kDa*  *NADH dehydrogenase (ubiquinone) 1 beta subcomplex, 5, 16kDa*  *NADH dehydrogenase (ubiquinone) 1 beta subcomplex, 6, 17kDa*  *NADH dehydrogenase (ubiquinone) 1 beta subcomplex, 8, 19kDa*  *NADH dehydrogenase (ubiquinone) 1 beta subcomplex, 9, 22kDa*  *NADH dehydrogenase (ubiquinone) Fe-S protein 4, 18kDa (NADH-coenzyme Q reductase)* | -0.820  -0.850  -0.676  -0.663  -0.678  -0.642  -0.733  -0.840  -0.608  -0.687  -0.703  -0.683 | 0.0002  0.001  0.02  0.03  0.02  0.03  0.01  0.001  0.05  0.02  0.02  0.02 |
|  | *PPA1* | *Pyrophosphatase (inorganic) 1* | -0.720 | 0.012 |
|  | *SLC25A4* | *Solute carrier family 25 (mitochondrial carrier; adenine nucleotide translocator), member 4* | -0.688 | 0.019 |
| ***ETC Complex IV*** | *COX3* | *Cytochrome c oxidase III* | -0.727 | 0.01 |
|  | *COX4I2*  *COX6C*  *COX7A2*  *COX7A2L*  *COX7C* | *Cytochrome c oxidase subunit IV isoform 2 (lung)*  *Cytochrome c oxidase subunit Vic*  *Cytochrome c oxidase subunit VIIa polypeptide 2*  *Cytochrome c oxidase subunit VIIa polypeptide 2 L*  *Cytochrome c oxidase subunit VIIc* | -0.642  -0.829  -0.711  -0.626  -0.647 | 0.03  0.002  0.01  0.04  0.03 |
| ***ETC Complex V*** | *ATP5J* | *ATP synthase, H^+^ transporting, mitochondrial Fo complex, subunit F6* | -0.717 | 0.01 |
|  | *ATP6*  *ATP6V0D2*  *ATP6V0E1* | *ATP6, ATPase, H^+^ transporting, lysosomal 38kDa, V0 subunit d2*  *ATP6V0D2, ATPase, H^+^ transporting, lysosomal 38kDa, V0 subunit d2*  *ATP6V0E1, ATPase, H^+^ transporting, lysosomal 9kDa, V0 subunit e1* | -0.860  -0.638  -0.801 | 0.0007  0.04  0.003 |
| ***TCA cycle*** | *FH* | *Fumarate hydratase* | -0.644 | 0.03 |
| ***Fatty acid biosynthesis*** | *ACACB* | *Acetyl-CoA carboxylase beta* | -0.607 | 0.02 |
| ***Alanine-aspartate glutamate metabolism*** | *NIT2* | *Nitrilase family member 2* | -0.641 | 0.034 |
| ***mTOR signaling*** | *RPS6*  *AKT3*  *BRAF* | *Ribosomal protein S6*  *V-akt murine thymoma viral oncogene homolog 3*  *B-Raf proto-oncogene, serine/threonine kinase* | -0.903  -0.720  -0.657 | 0.0001  0.01  0.03 |
|  |  |  |  |  |
|  |  | **Positive Correlations** |  |  |
|  | **Gene** | **Gene description** | **R-value** | **P-value** |
| ***Oxidative phosphorylation*** |  |  |  |  |
| ***ETC Complex V*** | ATP6V1C1 | ATPase, H^+^ transporting, lysosomal 42kDa, V1 subunit C1 | 0.604 | 0.05 |
| ***Pyruvate metabolism*** | *PKM*  *ALDH7A1* | PKM, pyruvate kinase, muscle  ALDH7A1, aldehyde dehydrogenase 7 family, member A1, lysine metabolism | 0.656  0.799 | 0.03  0.003 |
| ***Alanine-aspartate glutamate metabolism*** | *GLS2*  *ASL* | Glutaminase 2 (liver, mitochondrial)  Argininosuccinate lyase | 0.874  0.618 | 0.0004  0.043 |
| Source, R2: Genomics Analysis and Visualization Platform (http://r2.amc.nl). The genome samples used in this analysis are from activated CD4^+^ and CD8^+^ T cells isolated from tonsils of healthy human donors (n=11) and deposited in R2 by Eckerle and co-authors (ref. 18). | | | | |

| **Supplementary Table 4.** Primer sequences | | |
| --- | --- | --- |
| **Genotyping PCR primer sequences** | | |
| **Gene** | **Forward** | **Reverse** |
| *Bcat1* | 5’ GTCTGTGGAGGTCTCAAGTCAGCTTG 3’ | 5’ ATCCCAGAAGG TCACCCAAACAAAG 3’ |
| *Bcat2* | 5’ GTTCTCAAGGTGGTGGGTGT 3’ | 5’ TCCCTGGTGCCTGACACTAAA 3’ |
|  | - | 5’ AGAAGCCACAGGGGAAATGT 3’ |
| *CD4Cre* | 5’ GTTCTTTGTATATATTGAATGTTAGCC 3’ | - |
| *WT-CD4Cre* | - | 5’ TATGCTCTAAGGACAAGAATTGACA 3’ |
| *Mut-CD4Cre* | - | 5’ CTTTGCAGAGGGCTAACAGC 3’ |
| **RT-qPCR primer sequences** | | |
| **Gene** | **Forward** | **Reverse** |
| *Bcat1* | 5’ GTACATCAGAGCCTGGAAAGG 3’ | 5’ ACCTGCTGACAGCCATTCTC 3’ |
| *Bcat2* | 5’ TGGTCTGCACTACTCTCTGC 3’ | 5’ TTGTCAAAGTCTGGCAGGCA 3’ |
| *CD4Cre* | 5’ GTTCTTTGTATATATTGAATGTTAGCC 3’ | 5’ CTTTGCAGAGGGCTAACAGC 3’ |
| *Ef1a* | 5’ GACGCTGCTCAACACCATGGAC 3’ | 5’ CGGTACTGGCCAAACACCAG 3’ |

| **Supplementary Table 5.** Amino acid concentrations in T-cell medium at 0 h or in medium from unstimulated, TCR-stimulated or co-stimulated CD4^+^ T cells from WT and G-BCATm^KO^ mice after 24 h of growth | | | | | | | | | | | | | | | | | | | | | |
| --- | --- | --- | --- | --- | --- | --- | --- | --- | --- | --- | --- | --- | --- | --- | --- | --- | --- | --- | --- | --- | --- |
| **0 h 24 h 24 h 24 h** | | | | | | | | | | | | | | | | | | | | | |
| **Unstimulated cells TCR-stimulated cells Co-stimulated cells** | | | | | | | | | | | | | | | | | | | | | |
| **AA T-cell medium WT G-BCATm^KO^ WT G-BCATm^KO^  WT G-BCATm^KO^** | | | | | | | | | | | | | | | | | | | | | |
| **LEU** | 310 | ± | 9 | 338 | ± | 4^#^ | 339 | ± | 10^#^ | 251 | ± | 12^#^ | 299 | ± | 13 | 280 | ± | 12^#^ | 236 | ± | 38^#^ |
| **ILE** | 289 | ± | 11 | 323 | ± | 3^#^ | 324 | ± | 11^#^ | 236 | ± | 14 | 290 | ± | 18 | 289 | ± | 13 | 296 | ± | 17 |
| **VAL** | 158 | ± | 16 | 186 | ± | 5^#^ | 182 | ± | 4 | 118 | ± | 6# | 148 | ± | 14 | 142 | ± | 10 | 91 | ± | 9***^#^ |
| **PHE** | 87 | ± | 5 | 90 | ± | 2 | 90 | ± | 1 | 69 | ± | 7^#^ | 75 | ± | 2^#^ | 67 | ± | 1^#^ | 53 | ± | 3***^#^ |
| **TRP** | 23 | ± | 0 | 25 | ± | 1 | 23 | ± | 1 | 20 | ± | 1^#^ | 19 | ± | 1^#^ | 18 | ± | 1^#^ | 16 | ± | 2^#^ |
| **THR** | 136 | ± | 18 | 162 | ± | 3 | 159 | ± | 5 | 100 | ± | 7# | 137 | ± | 11** | 131 | ± | 12 | 84 | ± | 11*** |
| **HIS** | 62 | ± | 16 | 75 | ± | 2 | 74 | ± | 6 | 52 | ± | 5 | 53 | ± | 10 | 62 | ± | 2 | 53 | ± | 4 |
| **MET** | 69 | ± | 2 | 78 | ± | 2^#^ | 78 | ± | 2^#^ | 58 | ± | 5^#^ | 65 | ± | 2 | 57 | ± | 3^#^ | 47 | ± | 7^#^ |
| **LYS** | 182 | ± | 14 | 193 | ± | 4 | 198 | ± | 6 | 140 | ± | 1^#^ | 168 | ± | 6** | 152 | ± | 9^#^ | 116 | ± | 9***^#^ |
| **ARG** | 704 | ± | 56 | 728 | ± | 11 | 720 | ± | 13 | 616 | ± | 33^#^ | 705 | ± | 30 | 714 | ± | 34 | 582 | ± | 98 |
| **ORN** | 49 | ± | 5 | 64 | ± | 0^#^ | 69 | ± | 6^#^ | 62 | ± | 3^#^ | 65 | ± | 4^#^ | 76 | ± | 17^#^ | 60 | ± | 5 |
| **GLU** | 168 | ± | 14 | 267 | ± | 4^#^ | 297 | ± | 6*^#^ | 347 | ± | 22^#^ | 458 | ± | 28**^#^ | 483 | ± | 20^#^ | 381 | ± | 90^#^ |
| **GLN** | 2651 | ± | 135 | 2706 | ± | 38 | 2682 | ± | 56 | 2124 | ± | 279^#^ | 2387 | ± | 98^#^ | 2318 | ± | 110^#^ | 1833 | ± | 316^#^ |
| **ASP** | 127 | ± | 2 | 151 | ± | 1 | 152 | ± | 2 | 132 | ± | 8 | 175 | ± | 13** | 201 | ± | 12^#^ | 137 | ± | 39 |
| **ASN** | 256 | ± | 15 | 298 | ± | 5^#^ | 303 | ± | 1^#^ | 237 | ± | 38 | 282 | ± | 16 | 282 | ± | 11^#^ | 218 | ± | 51 |
| **ALA** | 77 | ± | 7 | 113 | ± | 1^#^ | 113 | ± | 1^#^ | 82 | ± | 10 | 98 | ± | 6^#^ | 96 | ± | 4^#^ | 72 | ± | 14 |
| **GLY** | 156 | ± | 2 | 247 | ± | 6^#^ | 236 | ± | 3*^#^ | 202 | ± | 9^#^ | 215 | ± | 1^#^ | 238 | ± | 26^#^ | 221 | ± | 14^#^ |
| **SER** | 200 | ± | 13 | 188 | ± | 5 | 191 | ± | 5 | 155 | ± | 20^#^ | 177 | ± | 15 | 176 | ± | 9^#^ | 127 | ± | 23***^#^ |
| **TYR** | 93 | ± | 8 | 102 | ± | 2 | 102 | ± | 2 | 77 | ± | 6^#^ | 87 | ± | 10 | 87 | ± | 5 | 66 | ± | 12***^#^ |
| **TAU** | 6 | ± | 0 | 4 | ± | 1^#^ | 5 | ± | 1^#^ | 13 | ± | 2^#^ | 13 | ± | 1^#^ | 14 | ± | 1^#^ | 12 | ± | 0^#^ |
| Amino acid (AA) concentrations are in µM. The experiment was repeated three independent times; shown values are the mean +/- SD of a single representative experiment, n=3 independent experiments each with n=3 internal repeats; **p* ≤ 0.05 as compared to un-stimulated WT T cells, ***p* ≤ 0.05 as compared to TCR-stimulated WT T cells, ****p* ≤0.05 as compared to co-stimulated WT T cells; ^#^*p* ≤0.05 as compared to T-cell medium at 0 h. G-BCATm^KO^, mouse model with a global deletion of BCATm. T-cell medium is defined as the growth medium added freshly to cell cultures at 0 h of cell seeding. | | | | | | | | | | | | | | | | | | | | | |

| **Supplementary Table 6.** Selected amino acid concentrations in cell pellets from un-stimulated, TCR-stimulated or co-stimulated CD4^+^ T cells from WT and G-BCATm^KO^ mice | | | | | | | | | | | | | | | | | | |
| --- | --- | --- | --- | --- | --- | --- | --- | --- | --- | --- | --- | --- | --- | --- | --- | --- | --- | --- |
| **24 h 24 h 24 h** | | | | | | | | | | | | | | | | | | |
| **Unstimulated cells TCR-stimulated cells Co-stimulated cells** | | | | | | | | | | | | | | | | | | |
| **AA WT G-BCATm^KO^ WT G-BCATm^KO^ WT G-BCATm^KO^** | | | | | | | | | | | | | | | | | | |
| **LEU** | 52 | ± | 1.8 | 66 | ± | 4.3* | 43 | ± | 1.4 | 57 | ± | 2.5** | 64 | ± | 3.3 | 77 | ± | 1.0*** |
| **ILE** | 49 | ± | 1.6 | 67 | ± | 1.3* | 45 | ± | 2.6 | 58 | ± | 3.0** | 65 | ± | 1.6 | 76 | ± | 2.8*** |
| **VAL** | 48 | ± | 0.9 | 64 | ± | 0.6* | 26 | ± | 2.9 | 32 | ± | 1.2** | 31 | ± | 0.4 | 39 | ± | 1.1*** |
| **PHE** | 25 | ± | 2 | 23 | ± | 3 | 17 | ± | 1 | 17 | ± | 1 | 22 | ± | 1 | 22 | ± | 1 |
| **TRP** | 7 | ± | 1 | 7 | ± | 1 | 6 | ± | 1 | 5 | ± | 2 | 6 | ± | 0 | 6 | ± | 0 |
| **THR** | 141 | ± | 28 | 151 | ± | 14 | 36 | ± | 13 | 51 | ± | 8 | 56 | ± | 7 | 74 | ± | 1*** |
| **HIS** | nd | ± | nd | nd | ± | nd | nd | ± | nd | nd | ± | nd | nd | ± | nd | nd | ± | nd |
| **MET** | 19 | ± | 2 | 16 | ± | 1 | 10 | ± | 1 | 9 | ± | 1 | 11 | ± | 1 | 11 | ± | 0 |
| **LYS** | 115 | ± | 11 | 94 | ± | 8 | 40 | ± | 7 | 45 | ± | 2 | 46 | ± | 3 | 54 | ± | 1*** |
| **ARG** | 153 | ± | 4.9 | 194 | ± | 2.9* | 158 | ± | 2.8 | 170 | ± | 3.7** | 182 | ± | 3.8 | 220 | ± | 1.2*** |
| **ORN** | 46 | ± | 1 | 40 | ± | 5 | 28 | ± | 3 | 25 | ± | 1 | 30 | ± | 2 | 25 | ± | 1*** |
| **ASP** | 3986 | ± | 663 | 5029 | ± | 615 | 360 | ± | 100 | 604 | ± | 42** | 469 | ± | 52 | 736 | ± | 11*** |
| **GLU** | 3005 | ± | 427 | 3341 | ± | 351 | 2284 | ± | 543 | 2860 | ± | 190 | 2717 | ± | 623 | 3015 | ± | 911 |
| **GLN** | 1462 | ± | 46 | 1990 | ± | 15* | 372 | ± | 68 | 506 | ± | 33** | 676 | ± | 77 | 859 | ± | 76*** |
| **ASN** | 261 | ± | 37 | 260 | ± | 20 | 152 | ± | 36 | 161 | ± | 9 | 243 | ± | 26 | 229 | ± | 2 |
| **ALA** | 273 | ± | 25 | 257 | ± | 22 | 286 | ± | 51 | 252 | ± | 14 | 418 | ± | 60 | 329 | ± | 1.8*** |
| **GLY** | 1889 | ± | 84 | 2000 | ± | 29 | 434 | ± | 12 | 311 | ± | 16** | 586 | ± | 27 | 480 | ± | 3*** |
| **SER** | 169 | ± | 20 | 165 | ± | 18 | 91 | ± | 14 | 87 | ± | 9 | 107 | ± | 10 | 93 | ± | 2 |
| **TYR** | 53 | ± | 4 | 49 | ± | 3 | 28 | ± | 4 | 30 | ± | 2 | 36 | ± | 3 | 36 | ± | 1 |
| **TAU** | 9838 | ± | 147* | 10782 | ± | 97* | 2251 | ± | 41 | 1971 | ± | 51** | 2930 | ± | 45 | 2726 | ± | 13*** |
| Amino acid (AA) concentrations were calculated as nmol/g wet pellet. The experiment was repeated three independent times; shown values are the mean +/- SD of a single representative experiment, n=3 independent experiments each with n=3 internal repeats; **p* ≤ 0.05 as compared to un-stimulated WT T cells, ***p* ≤ 0.05 as compared to TCR-stimulated WT T cells, ****p* ≤0.05 as compared to co-stimulated WT T cells; nd, not detected; G-BCATm^KO^, mouse model with a global deletion of BCATm. Leu, Ile, Val, Glu, Gln, and Arg are also included in Figure 1de. | | | | | | | | | | | | | | | | | | |

| **Supplementary Table 7.** Selected organ weights of male T-BCATm^fl/fl^ and T-BCATm^KO^ mice at the conclusion of the lymphoma tumor study | | | | |
| --- | --- | --- | --- | --- |
|  | | | | |
| T-BCATm^fl/fl^ T-BCATm^KO^ | | | | |
| Organ weight  [average ± standard error] | Vehicle | EL4-OVA | Vehicle | EL4-OVA |
| Spleen (g) | 0.069 ± 0.009 | 0.084 ± 0.005 | 0.064 ± 0.009 | 0.089 ± 0.008 |
| Thymus (g) | 0.053 ± 0.014 | 0.050 ± 0.007 | 0.042 ± 0.004 | 0.052 ± 0.007 |
| Heart (g) | 0.149 ± 0.006 | 0.175 ± 0.010 | 0.160 ± 0.018 | 0.154 ± 0.011 |
| Kidney (g) | 0.355 ± 0.015 | 0.380 ± 0.022 | 0.332 ± 0.025 | 0.355 ± 0.016 |
| Liver (g) | 1.338 ± 0.080 | 1.399 ± 0.040 | 1.316 ± 0.160 | 1.259 ± 0.040 * |
| Brain (g) | 0.363 ± 0.021 | 0.420 ± 0.013 | 0.422 ± 0.008 | 0.413 ± 0.010 |
| Lungs (g) | 0.181 ± 0.017 | 0.218 ± 0.015 | 0.165 ± 0.015 | 0.193 ± 0.013 |
| Male mice, age between 8-15 weeks, were subcutaneously injected with saline solution (vehicle) or 2.5x10^5^ EL4-OVA cells for 15 days. Values are shown as the average +/- SE, n=3 vehicle-injected mice/variant or n=8-12 EL4-OVA-injected mice /variant. **p* < 0.05 as compared with tumor-injected T-BCATm^fl/fl^ mice. The organ weights were normalized to 25 g body weight. | | | | |

| **Supplementary Table 8.** Selected organ weights of female T-BCATm^fl/fl^ and T-BCATm^KO^ mice at the conclusion of the lymphoma tumor study | | | | |
| --- | --- | --- | --- | --- |
|  | | | | |
| T-BCATm^fl/fl^ T-BCATm^KO^ | | | | |
| Organ weight  [average ± standard error] | Vehicle | EL4-OVA | Vehicle | EL4-OVA |
| Spleen (g) | 0.090 ± 0.008 | 0.095 ± 0.004 | 0.090 ± 0.005 | 0.095 ± 0.004 |
| Thymus (g) | 0.070 ± 0.014 | 0.052 ± 0.002 | 0.122 ± 0.036 | 0.054 ± 0.003* |
| Heart (g) | 0.140 ± 0.003 | 0.152± 0.017 | 0.180 ± 0.021^#^ | 0.127 ± 0.005* |
| Kidney (g) | 0.355 ± 0.015 | 0.380 ± 0.022 | 0.332 ± 0.025 | 0.355 ± 0.016 |
| Liver (g) | 1.458 ± 0.073 | 1.417 ± 0.053 | 1.505 ± 0.013 | 1.428 ± 0.051 |
| Brain (g) | 0.452 ± 0.043 | 0.497 ± 0.028 | 0.546 ± 0.076 | 0.468 ± 0.037 |
| Lungs (g) | 0.215 ± 0.031 | 0.203 ± 0.017 | 0.248 ± 0.015 | 0.190 ± 0.010* |
| Female mice, age between 9-15 weeks, were subcutaneously injected with saline solution (vehicle) or 2.5x10^5^ EL4-OVA cells for 15 days. Values are shown as the average +/- SE, n=3-5 vehicle-injected mice/variant or n=8-9 EL4-OVA-injected mice /variant. **p* < 0.05 as compared with tumor-injected T-BCATm^fl/fl^ mice. ^#^*p* < 0.05 as compared with vehicle-injected T-BCATm^fl/fl^ mice. The organ weights were normalized to 25 g body weight. | | | | |
