# Supplementary material for: Blocking mitochondrial leucine transamination enhances T-cell activation and improves T-cell immunity against OVA-producing EL4 lymphoma
Source: Br J Cancer. 2026 May 5;135(3):406–17. doi: 10.1038/s41416-026-03455-5 (PMC13372810; doi:10.1038/s41416-026-03455-5)
Supplement: Supplementary file 2 — Supplementary Figures 1-5 [file 41416_2026_3455_MOESM2_ESM.pdf]

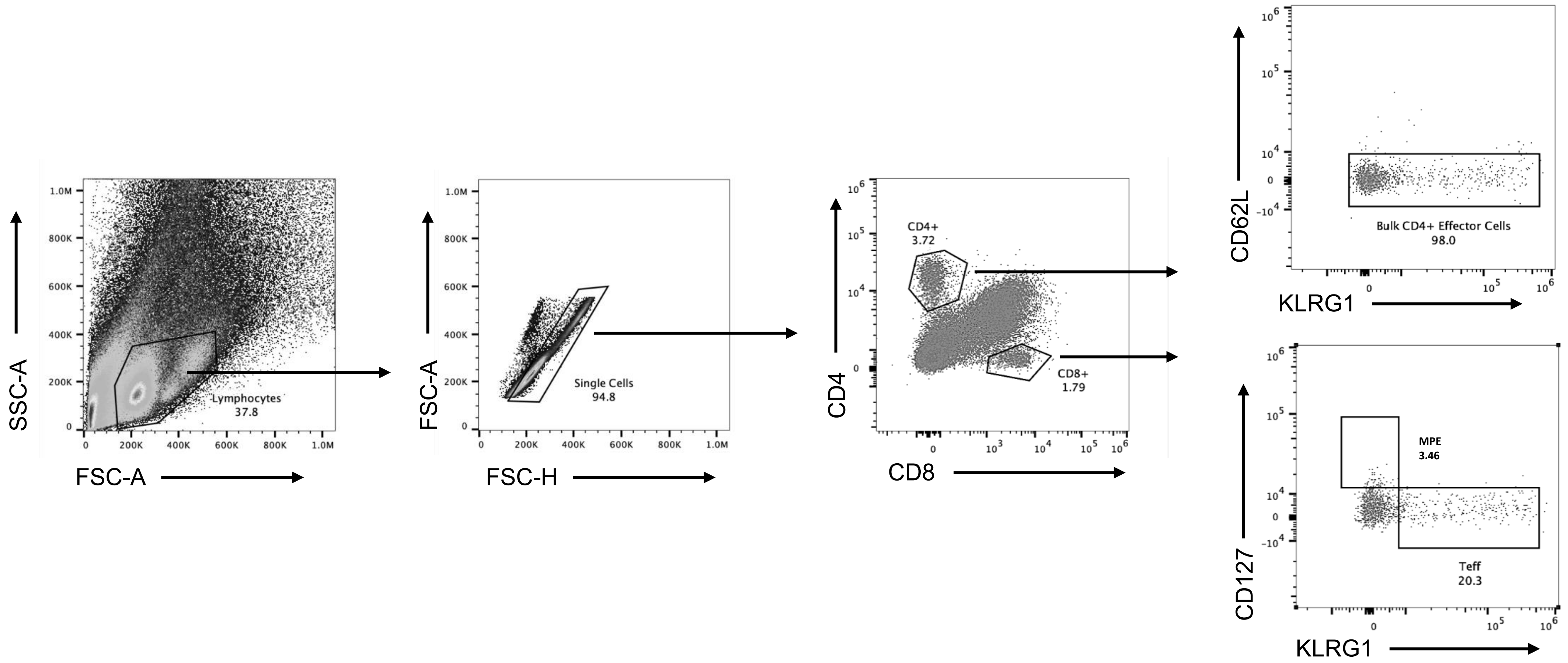

Supplementary Figure 1. Gating Strategy used to identify CD4⁺ and CD8⁺ T cell populations in the spleen, LN, thymus, or tumors (EL4-OVA induced mice only) isolated from naïve or EL4-OVA inoculated T-BCATm^{KO} mice and their respective controls to quantify the percentage of CD4⁺ and CD8⁺ bulk effector, terminal effector (Teff) and memory precursor effector (MPE) T cells.

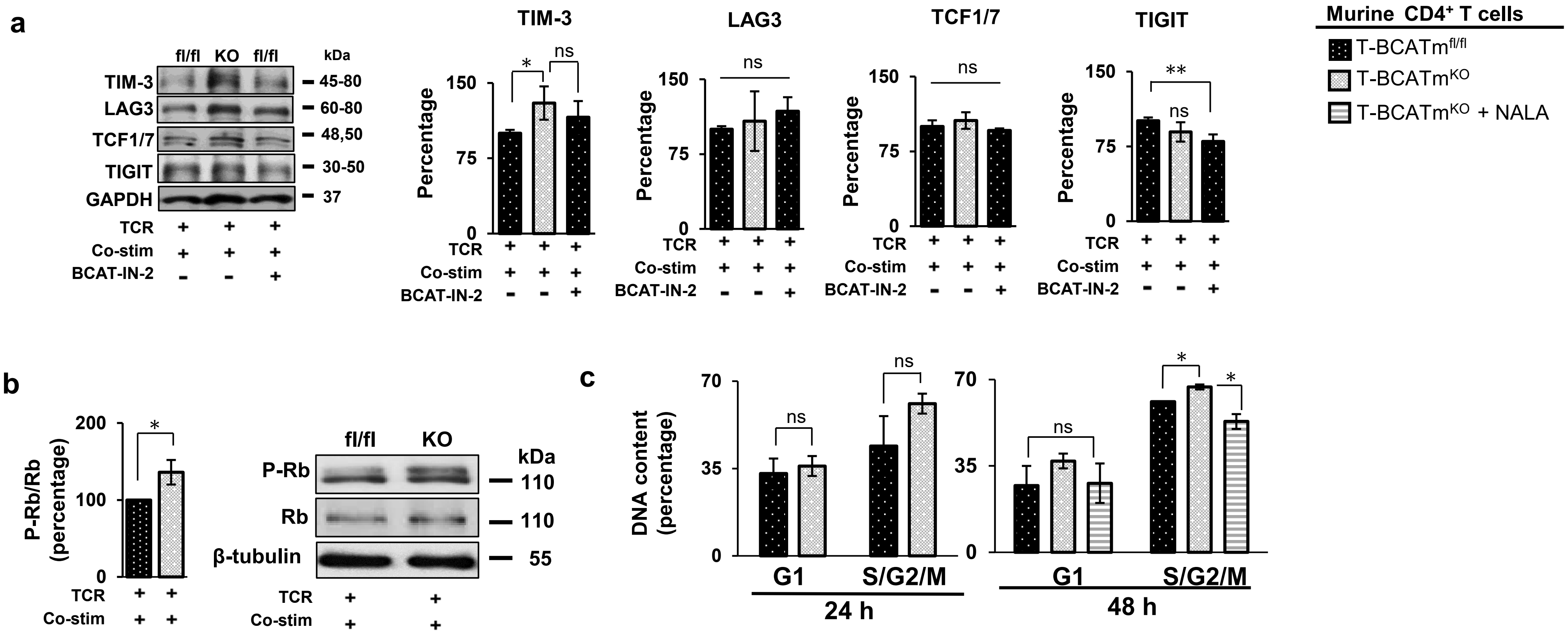

Supplementary Figure 2. a-c Previously activated CD4$^+$ T cells from T-BCATm$^{fl/fl}$ (fl/fl) and T-BCATm^{KO} (KO) mice were re-challenged with anti-CD3/CD28 (co-stimulation) for 24-72 h. Some of the cells were treated with 50 µM BCAT-IN-2 to inhibit BCATm enzyme activity (**a**) or 20 mM NALA to mimic leucine depletion (refer to **c**, T-BCATm^{KO} at 48 h) (n=16-18 mice/variant). In **a**, protein expression of makers of T cell exhaustion, TIM-3, LAG3, TCF1/7, and TIGIT as assessed by western botting. GAPDH or β-tubulin were used as a loading control. Image J was used to quantify the relative band intensity of these markers as normalized to GAPDH. Representative Western blotting images of 2 sets of n=3 mice/variant are shown. In **b**, protein expression of retinoblastoma (Rb) and its phosphorylation (P) state. Image J was used to quantify the relative band intensity of Rb and P-Rb and the ratio of P-Rb/Rb is presented as percentage of T cells from T-BCATm$^{fl/fl}$ mice. The Western blot images were representative of 3 independent experiments. In **c**, co-stimulated cells were subjected to propidium iodide to measure the DNA content in G1 or combined S/G2/M phases of the cell cycle (n=6 mice/variant). For all panels, average +/- SEM of mixed sex. Statistical significance as determined by a two-tailed Student's t-test: *p<0.05, **p<0.01, ***p<0.001 or ns= no significance.

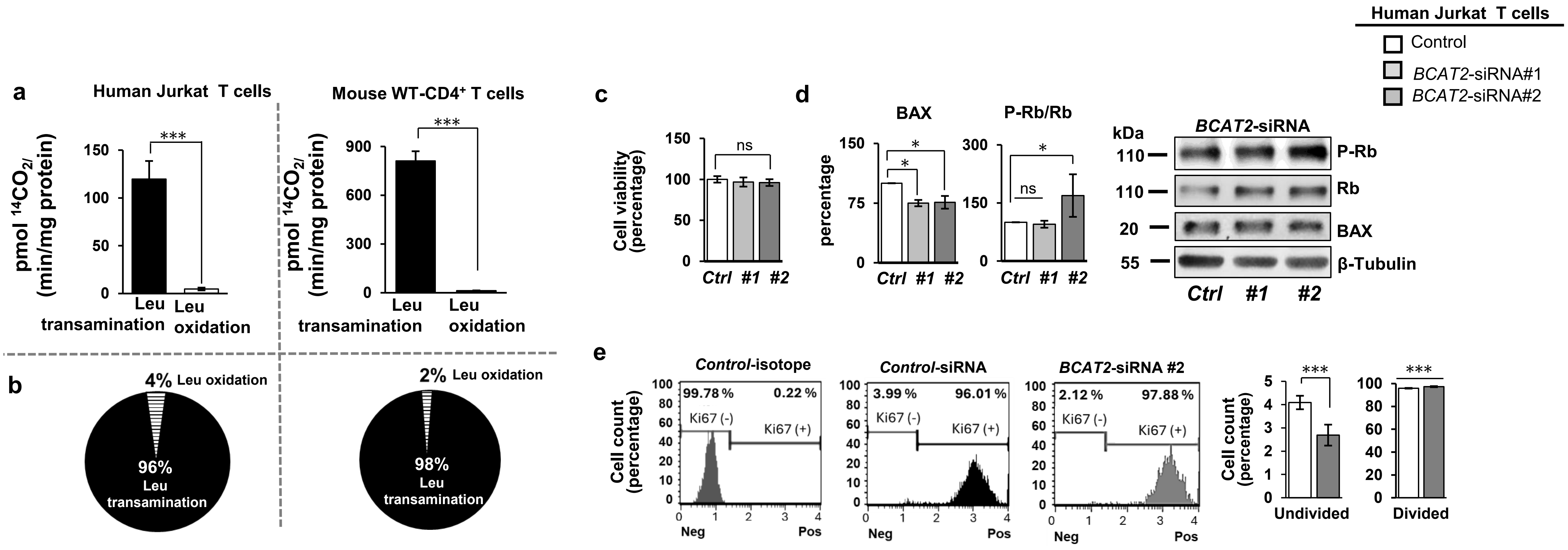

Supplementary Figure 3. a-b. Comparison of leucine transamination and oxidation in human Jurkat T cells and murine CD4+ T cells. In **a,** untreated human Jurkat T cells in their exponential phase of growth (n=3 independent cultures) and anti-CD3/CD28 co-stimulated murine wild-type (WT) CD4+ T cells (n≥ 6 mice) were fed ^{14}C-leucine during a leucine metabolic assays to determine the rates of leucine transamination and oxidation in each cell type, as described in the Methods. In **b**, the fractions of leucine transamination and oxidation are expressed as a percentage of the total leucine catabolism for each cell type in the accompanying pie charts. **c-e**. Human Jurkat T cells subjected to siRNA using 200 nM of control-siRNA (Ctrl) or *BCAT2*-siRNA#1 and #2 for 72 h (n≥ 3 independent cultures). In **c**, cell viability measured by the MTT assay. In **d**, protein expression of BAX and retinoblastoma (Rb) and Rb phosphorylation (P) state as assessed by western blotting. Image J was used to quantify the relative band intensity of BAX, Rb and P-Rb and the ratio of P-Rb/Rb is presented as percentage of control cells. β-tubulin was used to normalize BAX and as a loading control. The Western blotting images were representative of 3 independent experiments. In **e**, cell proliferation measured using the proliferative marker Ki67 and shown with representative charts and average results expressed as percentage from control cells. For all panels, data are average +/- SEM. Statistical significance as determined by a two-tailed Student's t-test: *p<0.05, **p<0.01, ***p<0.001 or ns= no significance.

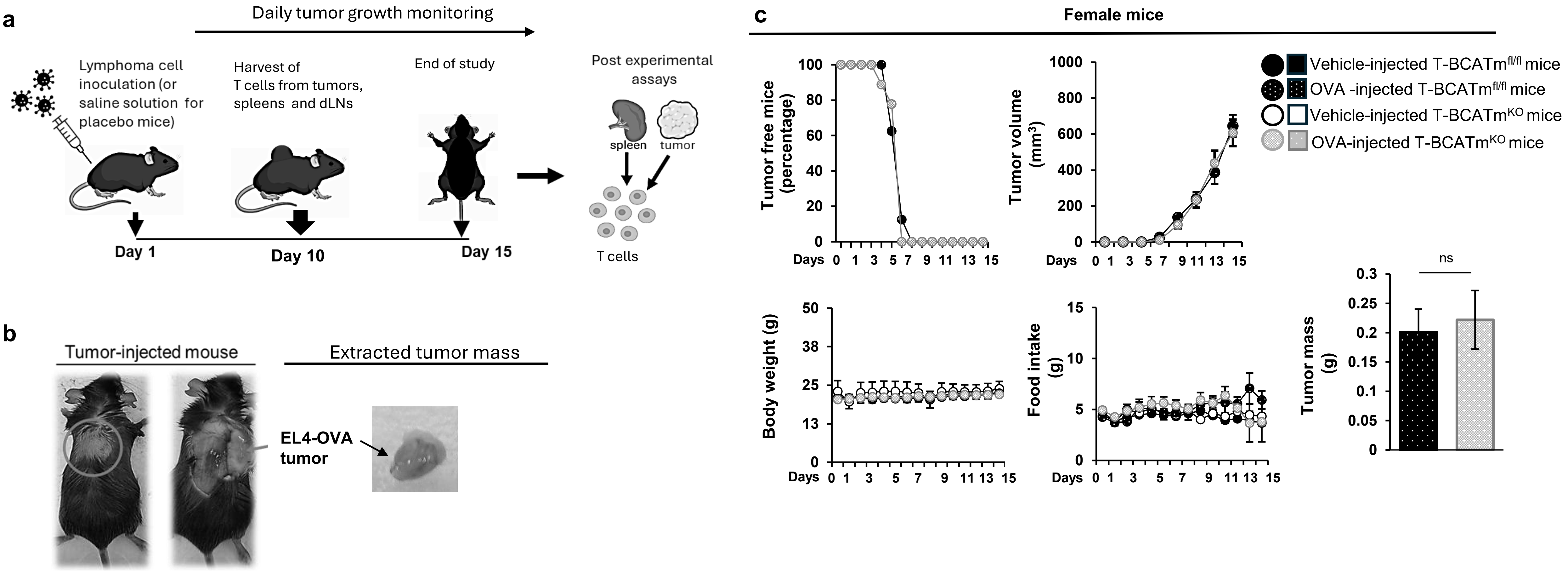

Supplementary Figure 4. In **a**, experimental design of the *in vivo* tumor studies. In **b**, a representative image of a tumor-injected mouse taken at conclusion of the tumor study. A flat appearing lump on the back of the tumor-injected mouse (circled with red line) was first detected around days 5-6.The EL4-OVA tumor appeared as a solid tumor with white–pink coloration. In **c**, female T-BCATm$^{fl/fl}$ and T-BCATm^{KO} mice that were vehicle (n=3-5 placebo mice) or tumor (2.5×10^5 EL4-OVA lymphoma, n=9 mice /genotype) injected were monitored for 15 days for tumor growth. The graphs show tumor free mice, tumor development over time, body weight, food intake, and tumor masses measured after mouse sacrifice. For all panels, data are average +/- SEM. Statistical significance as determined by a two-tailed Student's t-test: ns, not significant.

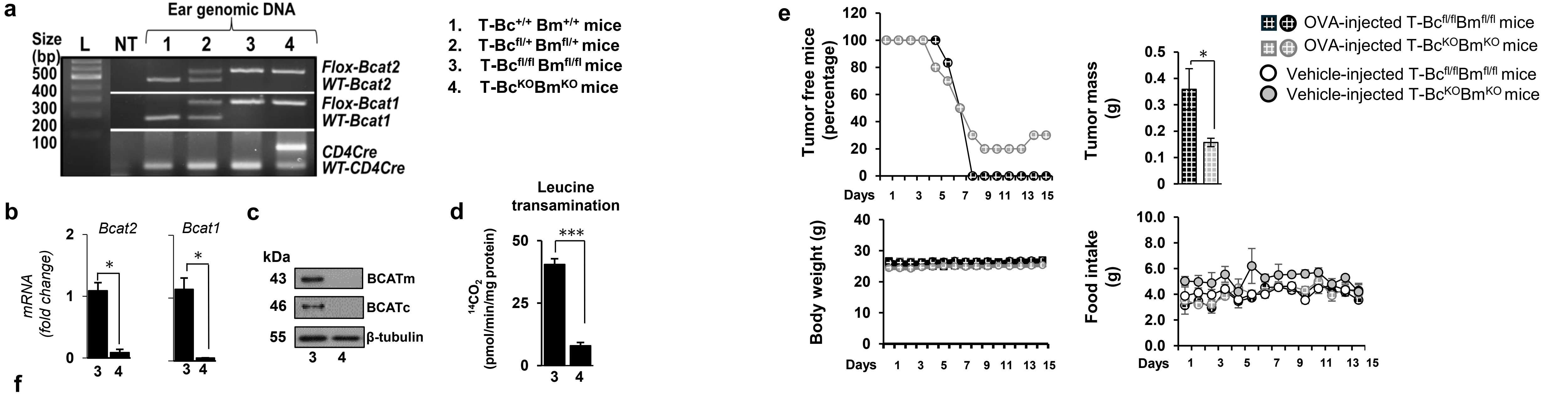

f

Selected organ weights of male T-Bc$^{fl/fl}$Bm$^{fl/fl}$ and T-Bc^{KO}Bm^{KO} mice at the conclusion of the lymphoma tumor study.

	T-Bc$^{fl/fl}$Bm$^{fl/fl}$		T-Bc^{KO}Bm^{KO}	
Organ weight [average ± standard error]	Vehicle (n=4)	EL4-OVA (n=6)	Vehicle (n=4)	EL4-OVA (n=8-10)
Spleen (g)	0.069 ± 0.004	0.078 ± 0.002	0.063 ± 0.003	0.074 ± 0.002**
Thymus (g)	0.043 ± 0.002	0.048 ± 0.007	0.027 ± 0.004*	0.059 ± 0.006**
Heart (g)	0.144 ± 0.012	0.154 ± 0.014	0.126 ± 0.006	0.147 ± 0.010
Kidney (g)	0.337 ± 0.012	0.350 ± 0.006	0.342 ± 0.026	0.369 ± 0.011
Liver (g)	1.267 ± 0.041	1.394 ± 0.028*	1.323 ± 0.030	1.399 ± 0.024
Brain (g)	0.394 ± 0.021	0.415 ± 0.011	0.407 ± 0.039	0.393 ± 0.012
Lungs (g)	0.198 ± 0.005	0.195 ± 0.020	0.180 ± 0.012	0.207 ± 0.014

*p<0.05 compared to T-Bc$^{fl/fl}$Bm$^{fl/fl}$ vehicle, **p<0.05 compared to T-Bc^{KO}Bm^{KO} vehicle. Organs normalized to 25 g body weight.

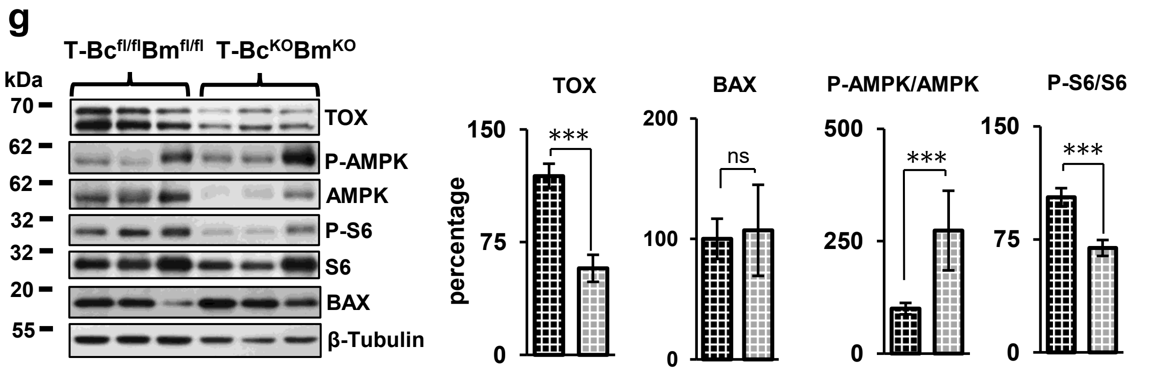

Supplementary Figure 5. In a-d, characterization of mice carrying a double (combined) deletion of *Bcat1&2* in T cells. In **a**, PCR of genomic ear DNA showing WT and floxed alleles of *Bcat1, Bcat2* and CD4Cre (maintained in a hemizygous state). A separate WT band was amplified to confirm this state (WT-CD4Cre). In **b**, *Bcat1* and *Bcat2* mRNA in T cells from T-Bc^{KO}Bm^{KO} mice (“4”) compared to T cells from control T-Bc$^{fl/fl}$Bm$^{fl/fl}$ mice (“3”). In **c**, western blotting of CD4^{+}T cell lysates verifying a loss of protein expression of BCATc and BCATm in T-Bc^{KO}Bm^{KO} mice (“4”). In **d**, ^{14}C-leucine metabolic assay showing severe reduction in leucine transamination in the absence of BCATc and BCATm in activated CD4^{+} T cells from T-Bc^{KO}Bm^{KO} mice (“4”) (n ≥ 4 mice/group). In **e**, lymphoma tumor study using male T-Bc$^{fl/fl}$Bm$^{fl/fl}$ (n=6) and T-Bc^{KO}Bm^{KO} (n=8) mice, age between 8-12 weeks old, s.c. inoculated with 2.5x10^{5} EL4-OVA cells. The graphs are showing tumor free mice, average tumor mass, body weight and food intake. In **f**. Selected organ weights. In **g**, Western blotting of tumors showing changes in the expression of TOX, BAX, AMPK, and S6 along with the phosphorylation (P-AMPK, P-S6) states. Image J was used to quantify the relative band intensity of TOX and BAX as normalized to β-tubulin or the ratio P-AMPK/ AMPK or P-S6/S6, respectively. Western blotting images were representative of 6 or more tumors. Legend [+/+ wild type alleles], [fl/+ heterozygous floxed and wild type alleles], [fl/fl, homozygous floxed alleles], [KO, absence of floxed alleles from T cells]. In all graphs, data represent mean ± SEM, *p<0.05, **p<0.01, ***p<0.001, ns, not significant.
