# Supplementary material for: Blocking mitochondrial leucine transamination enhances T-cell activation and improves T-cell immunity against OVA-producing EL4 lymphoma
Source: Br J Cancer. 2026 May 5;135(3):406–17. doi: 10.1038/s41416-026-03455-5 (PMC13372810; doi:10.1038/s41416-026-03455-5)
Supplement: Supplementary file 3 — Supplementary Information [file 41416_2026_3455_MOESM3_ESM.docx]

**Supplementary Information**

**ARRIVE 2.0**

***Essential 10***

**1.Study design**

This study relied on transgenic mouse models, which were used to explore lymphoma in ways impossible in humans. More specifically, the mouse models enabled us to test the metabolic reprograming of T cells as it occurred with the multitude of interactions in the whole animal when a single metabolic gene (***Bcat1 or Bcat2)*** or a combination of 2 metabolic genes (***Bcat1*** and ***Bcat2***) were removed from the T cell genome. This resulted in a loss of function of BCATm (since it is encoded by *Bcat2*) or BCATc (since it is encoded by *Bcat1*) or a combined loss of function of BCATm and BCATc in double and single positive CD4^+^ and CD8^+^ T cells of the transgenic mouse models. We characterized the effect of a loss of function of BCATm (and when applicable BCATc) in T cells using a variety of *in vitro* and *in vivo* assays as described in the Methods and Results sections. Below are schematics of experimental design during the *in vitro* and *in vivo* studies using mouse T cells along with additional information about the experimental groups in the accompanying tables. These studies were verified partially with human T cells or compared to publicly available genomic information from T cell datasets of human healthy donors and patients.

- 1. **
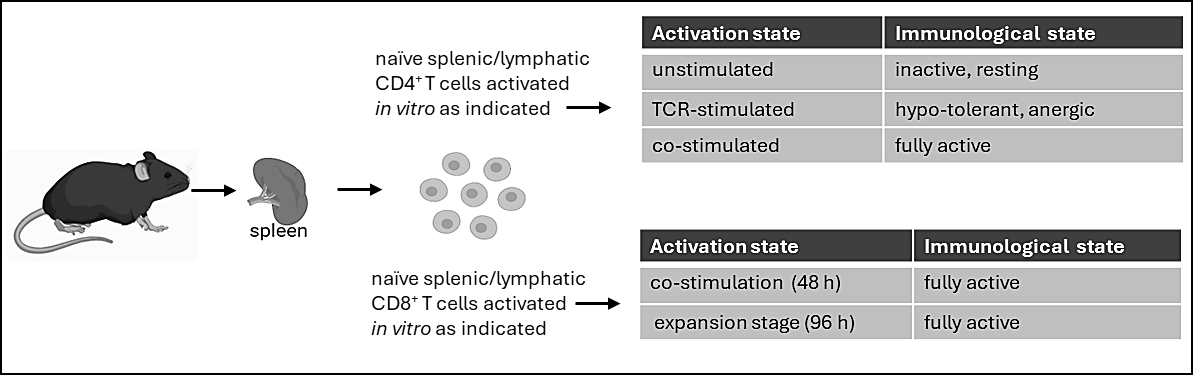
*In vitro* experiments**

| **Experimental animal groups during *in vitro* experiments.** | | |  |  |  |
| --- | --- | --- | --- | --- | --- |
| **Variants (including controls)** | **Mouse model** | **T cells** | **Sex** | **Total sample size** | **Age range (weeks)** |
| *Unstimulated/TCR or co-stimulated* | *WT* | *CD4^+^ T cells* | *mixed* | *n=18* | *12-22* |
| *Unstimulated/TCR or co-stimulated* | *Global BCATmKO* | *CD4^+^ T cells* | *mixed* | *n=18* | *12-22* |
|  |  |  |  |  |  |
| *Co-stimulated* | *T-BCATm^fl/fl^* | *CD4^+^ T cells* | *mixed* | *n=22* | *12-23* |
| *Co-stimulated +/- BCAT-IN-2* | *T-BCATm^fl/fl^* | *CD4^+^ T cells* | *mixed* | *n=6* | *12-23* |
| *Co-stimulated +/- NALA* | *T-BCATm^KO^* | *CD4^+^ T cells* | *mixed* | *n=24* | *15-23* |
| *Co-stimulated {48 hr}, Expanded {96 hr}* | *T-BCATm^fl/fl^* | *CD8^+^ T cells* | *mixed* | *n=28* | *10-20* |
| *Co-stimulated {48 hr}, Expanded {96 hr}* | *T-BCATm^KO^* | *CD8^+^ T cells* | *mixed* | *n=28* | *10-20* |
| *Co-stimulated {48 hr}, Expanded {96 hr}* | *T-Bc^fl/fl^Bm^fl/fl^* | *CD8^+^ T cells* | *mixed* | *n=19* | *13-20* |
| *Co-stimulated {48 hr}, Expanded {96 hr}* | *T-Bc^KO^Bm^KO^* | *CD8^+^ T cells* | *mixed* | *n=19* | *13-20* |

- 1. ***. In vivo* experiments**

**
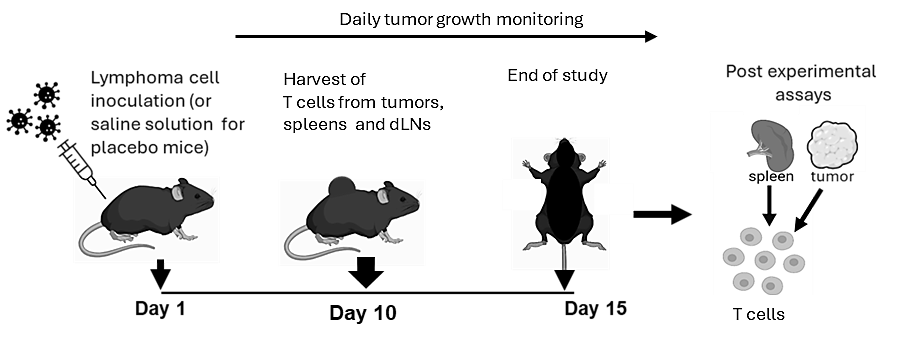
**

| **Experimental animal groups and lymphoma cell inoculation** | | |  |  |  |
| --- | --- | --- | --- | --- | --- |
| **Variants (sub-groups)** | **Mouse model** | **EL4-OVA cell inoculation** | **Sex** | **Sample size** | **Age range (weeks)** |
| *vehicle* | *T-BCATm^fl/fl^* | *no cells (PBS only)* | *male* | *n=3* | *10-15* |
| *tumor injected* | *T-BCATm^fl/fl^* | *2.5x10^5^ cells in PBS* | *male* | *n=12* | *9-12* |
| *vehicle* | *T-BCATm^KO^* | *no cells (PBS only)* | *male* | *n=3* | *11-14* |
| *tumor injected* | *T-BCATm^KO^* | *2.5x10^5^ cells in PBS* | *male* | *n=9* | *8-12* |
|  |  |  |  |  |  |
| *vehicle* | *T-BCATm^fl/fl^* | *no cells (PBS only)* | *female* | *n=5* | *11-14* |
| *tumor injected* | *T-BCATm^fl/fl^* | *2.5x10^5^ cells in PBS* | *female* | *n=8* | *11-13* |
| *vehicle* | *T-BCATm^KO^* | *no cells (PBS only)* | *female* | *n=5* | *9-15* |
| *tumor injected* | *T-BCATm^KO^* | *2.5x10^5^ cells in PBS* | *female* | *n=9* | *11-13* |
|  |  |  |  |  |  |
| *tumor injected* | *T-BCATc^fl/fl^* | *2.5x10^5^ cells in PBS* | *male* | *n=8* | *11-13* |
| *tumor injected* | *T-BCATc^KO^* | *2.5x10^5^ cells in PBS* | *male* | *n=8* | *11-13* |
|  |  |  |  |  |  |
| *vehicle* | *T-Bc^fl/fl^Bm^fl/fl^* | *no cells (PBS only)* | *male* | *n=4* | *12* |
| *tumor injected* | *T-Bc^fl/fl^Bm^fl/fl^* | *2.5x10^5^ cells in PBS* | *male* | *n=6* | *8-12* |
| *vehicle* | *T-Bc^KO^Bm^KO^* | *no cells (PBS only)* | *male* | *n=4* | *12* |
| *tumor injected* | *T-Bc^KO^Bm^KO^* | *2.5x10^5^ cells in PBS* | *male* | *n=8-10* | *8-12* |
|  |  |  |  |  |  |

1. **Sample size**
   1. **For *in vitro* studies**. The number of T cells, and subsequently the sample size, varied depending on how much material it was possible to obtain to ensure statistically significant results from 3 independent experiments. Harvest of one mouse spleen and lymph nodes yields ≥ 100x10^6^ splenocytes. The mouse spleen contains ~20% T cells. After purification, up to 10x10^6^ naïve T cells from a single mouse could be recovered by the method used in our laboratory. Depending on the experiment, between 5-50x10^6^ T cells/variant were needed. Thus, material from 2 or more mice/variant was pooled during a single experiment to increase total yield and the total sample size per experimental group is shown in the table under 1.1 above.
   2. **For *in vivo* tumor studies (tumor-injected mice)**. Based on a previous analogous tumor challenge performed by our research group, we reasonably assumed that the standard deviation of the tumor volume measurements would be at a magnitude of 33% of the mean tumor volume of the control mice. The effect of the control mice on the mean tumor volume was expected to be reduced by 25% and 50% in the single (T-BCATm^KO^  or T-BCATc^KO^) and double (T-Bc^KO^Bm^KO^) mice, respectively. Tumor injection was expected to cause an increase (at the magnitude of 20% of the mean tumor volume of control mice) compared to the vehicle injection. Therefore, we estimated a sample size of 15 mice per experimental group (mixed sex) to have at least 81% power at 5% significance to detect the assumed tumor volume changes above. The actual sample size used per experimental group [variant] is indicated in the table under 1.2 above.
2. **Inclusion and exclusion criteria for the *in vivo* tumor studies**
   - 1. **Inclusion criteria**
     2. Age between 8-15 weeks (young adult age).
     3. Sex, males, and females. Female outcomes were reported only for the T-BCATm^KO^ colony in this study.
     4. Immunocompetent mouse strain.
     5. Use of the C57BL/6 strain since the transgenic models were created under this strain.
     6. Lymphoma cells are syngeneic with the C57BL/6 strain.
   1. **Exclusion criteria**
      1. Mice experiencing difficulties reaching for food and losing more than 20% of their original body weight.
      2. Mice with abnormal behavior (aggressiveness, barbering).
      3. Mice with altered healthy status from birth (runts, hunched posture, malocclusions, hydrocephalus). ** excluded also from in vitro experiments.*
      4. Mice bearing tumors with ulcerations.
      5. Mice with excessive scratching around the site of tumor inoculation.
      6. Mice that underwent unsuccessful lymphoma cell inoculation due to human error.
      7. Mice underwent unsuccessful tumor measurements over time due to human or technical errors.
      8. Mice bearing tumors exceeding 10% of the body weight or volumes bigger than 2000 mm^3^.
3. **Randomization**
   1. All mice originated from the same genetic background (C57BL/6) and were maintained in the same animal care facility. Age-matched transgenic mice, and their littermate controls were genotyped using both sexes at regular intervals. Prior to experiments, mice were allocated to experimental groups (control versus knockout) on a random principle driven by the Mendelian inheritance if they did not meet the exclusion criteria.
   2. Cofounder effects- while a specific strategy was not developed to control for this, animal cages and measurements varied in location and order each time a new set of sub-groups was studied due to the nature of the experiments (multiple studies carried).
4. **Blinding/masking**
   1. For each tumor study, between two and three different investigators were involved: the first investigator (the PI) administered the cancer cell inoculation based on a randomization table prepared in advance. Since all mice received the same type and number of cancer cells, this step was not blinded. A second investigator (graduate student, blinded) was responsible for the anesthetic procedure and shaving of the mice, whereas a third investigator (graduate or doctoral student) performed the daily monitoring of body weight changes, food intake and tumor growth using a table with animal IDs but without indication of the animal genotype. Thus, the third investigator was blinded. The stage of data analysis was not blinded.
   2. The *in vitro* experiments were blinded prior to the assays where the mouse phenotype, mouse IDs, or treatments were coded with generic labels (e.g., Group A, Group B) instead of treatment/control or were assigned random numbers starting with “1”.
5. **Outcome measures**
   1. Explained in detail in the Methods and Results sections of the manuscript.
6. **Statistical methods**
   1. This report used the Student's t-test, the One-way ANOVA, and the Bartlett's test. Based on the purpose of each of these tests and the nature/number of compared variants, the selected tests were justified as appropriate to use. The Student's t-test was used to determine if the difference between the average of two variants was statistically significant when the ***p value*** was set at ≤ 0.05. The data points collected for each variant met the assumption for normal two tailed distribution (tails=2) and each two variants with equal number of data points [or sample size (n) being the same], were determined to have equal variance (homoscedastic, type=2). This was confirmed by using Bartlett’s test for homogeneity of variances. For comparison of multiple groups of variants or variants with unequal but similar sample size (for example, number of mice engaged in *in vivo* tumor studies), the One-way ANOVA test was used instead. Additional information on the statistical analysis is provided in the main body (Methods Section) of the manuscript.
7. **Experimental animals**
   1. **Species, strain, sex, weight, and age of animals**
      1. C57BL/6, males and females, age range between 8-23 weeks for all experiments. If animal age range was different during a specific experiment, this was indicated in the figure legend. Weight was measured prior to animal sacrifice for *in vitro* experiments or daily during the *in vivo* tumor studies (shown in the results section).
      2. Animal numbers during experiments (refer to “1” above) and the main body of the manuscript.
8. **Experimental procedures**

9.1. Refer to the main body (“Methods section”) of the manuscript.

1. **Results**

10.1. Refer to the main body (“Results section”) of the manuscript.

***Recommended set***

1. **Abstract**

11.1. Refer to the main body (“Abstract section”) of the manuscript.

1. **Background**

12.1. Refer to the main body of the manuscript.

1. **Objectives**

13.1. Refer to page 3 of the manuscript.

1. **Ethical statement**
   1. Des Moines University’s Animal Care and Use Program is fully accredited by the Association for Assessment and Accreditation of Laboratory Animal Care, International (AAALAC) and compliant with Public Health Service requirements (assurance identification number D16-00432 (A3732-01)) and the Animal Welfare Act regulations. The animal studies, associated with this manuscript, were approved under IACUC IDs 2023-01 and 2024-06-01 to E.A.A.
2. **Housing and husbandry**
   1. Mice were kept in ventilated solid bottom cages (NexGen Max IVC cage and rack system, Allentown, UK) with temperature between 20 °C to 26 °C, humidity between 30% and 70%, and a 12/12-hour light/dark cycle. Mice were offered a standardized mouse diet (Teklab diet, Iontiv, cat# 7012), with crude protein, not less than 19%; crude fat, not less than 5%, crude fiber, not more than 5%) and provided drinking water ad libitum (Municipal water, autoclaved before use, or gel packs from HydroGel 98% pure water for pups (ClearH2O, Inc., Westbrook, ME)). Water quality control, including monitoring contaminants, was performed by the Des Moines Water Works Department. Microbial surveys of the water were conducted every three months. Same sex littermates were housed together at the maximum density of five mice from the same litter and sex following weaning. Thus, mice were provided with their social environment before being taken for experiments. However, during the *in vivo* tumor studies, mice were housed individually to avoid fighting, barbering, scratching tumors, as well as monitoring food and water intake on individual level. *Enrichment items included* corn cob bedding (Teklab, Inotiv cat# 7092) for nesting, igloos, (Bio-Serv, cat# K3327), cardboard rolls and paper towels. Vermin control was managed by RiddX Pest Control every three months. Comprehensive PCR evaluation of sentinel mice was performed biannually by IDEXX BioAnalytics (Columbia, MO).
3. **Animal care and monitoring**
   1. Mouse handling was done using a combination of techniques, such as tail handling, cupping, and tunnel handling. Cupping and tunnel handling were part of the refined techniques used to reduce distress during tumor studies.
   2. Anesthesia (tumor studies). A small rodent anesthesia vaporizer (Viking Medical) was used to anesthetize the mice prior to lymphoma cell inoculation. Each animal was placed in an induction chamber, supplied with a mixture of oxygen and isoflurane (1.5- 2% at a flow rate of 0.4-0.8 liter/min), until lightly sedated. Next, the animal was taken out of the chamber and placed on a warm pad (Kent, Scientific) at which point the animal was maintained on isoflurane inhaled via a tube placed near the external nares of the animal. Level of anesthesia was determined by the lack of response to a painful stimulus (e.g. toe pinch reflex). Once the level of sedation was deemed adequate, a small area from the back of the animal was shaved with a mouse clipper followed by cleaning the area with a sterile alcohol prep pad and s.c. injection of the lymphoma cells using a sterile needle (23G) attached to 1 ml syringe.
   3. Background information on the mouse lymphoma strain used during the *in vivo* animal studies in this report. The murine EG7 cells, referred here as to the “lymphoma cells” were purchased from ATCC (Manassas, VA, EL4-OVA, Cat # CRL-2113) and represent T lymphoblasts originally derived from the C57BL/6 (H-2 b) mouse lymphoma cell line EL4 (ATCC Cat # TIB-39). To create the EL4-OVA strain, the EL4 cells were transfected with the plasmid pAc-neo-OVA, which carries a single copy of the chicken ovalbumin (OVA) mRNA and the neomycin (G418) resistance gene. The EL4-OVA cells synthesize and secrete OVA constitutively and are thus inherently immunogenic (PMID: 3261634). While EL4-OVA cells represent hematological malignancy, they form well localized solid tumors when subcutaneously implanted in mice (refer to Supplementary Figure B).
   4. Lymphoma cell inoculation. All mice were able to recover after the lymphoma cell inoculation without being distressed. The lymphoma cells were prepared under aseptic conditions. They were washed three times in sterile PBS buffer, were contamination free, and originated from frozen stocks in their 1^st^ to 2^nd^ passage relative to initial stock from the respective vendor as indicated in the Methods section.
   5. Growing tumors. Because the lymphoma cells were injected subcutaneously on the mouse lower back, the tumors grew under the skin and did not interfere with the function of vital organs, such as the lungs or digestive tract. The tumors did not impact the mouse survival or cause the development of metastasis for the proposed period of growth (up to 15 days).
   6. Tumor-bearing mice. The research team inspected daily for tumor growth. None of the tumors reached 2000 mm^3^ volume due to the presence of the OVA antigen, which made them more “visible” to the immune cells.
   7. Food and water. Because of their location, the tumors did not interfere with locomotion, so that the mice were unable to eat or drink.
   8. Analgesia. No mice were given analgesia. However, the protocol was approved for administering carprofen 4-5 mg/kg if mice were to experience distress.
   9. Cage change. To reduce/eliminate unnecessary distress, cage change was prohibited for all mice for the duration of the tumor study (15 days).
   10. Humane endpoints. All mice completed the tumor studies on day 15 post lymphoma cell inoculation as determined by the experimental design. Because these mice never developed tumors larger than 2000 mm^3^, earlier termination was not applicable.
   11. Any change in animal behavior, health, and/or number, was recorded no later than 24 hours post observation. *Of note, no adverse events were observed during these studies.*
4. **Interpretation/scientific implications**
   1. This is addressed in detail in the “Discussion Section” of the manuscript.
5. **Generalizability/translation**
   1. This is addressed in a statement in the “Discussion Section” of the manuscript.
6. **Protocol registration**
   1. This study was not pre-registered. The animal protocols were approved by the IACUC committee of Virginia Tech and Des Moines University (refer to “14” above) and are currently under embargo along with the transgenic mouse lines as approved by the funding received by NIH-NCI (1R15CA249796-01A1 awarded to E.A.A).
7. **Data access**
   1. This manuscript did not generate datasets.
8. **Declaration of interests**
   1. The authors have declared no competing interests.
